# Electrostatic anti-CD33-antibody–protamine nanocarriers as platform for a targeted treatment of acute myeloid leukemia

**DOI:** 10.1186/s13045-022-01390-5

**Published:** 2022-12-01

**Authors:** Nicole Bäumer, Annika Scheller, Lisa Wittmann, Andreas Faust, Mara Apel, Subbaiah Chary Nimmagadda, Christiane Geyer, Katharina Grunert, Neele Kellmann, Matthias Peipp, Sareetha Kailayangiri, Matias Ezequiel Gutierrez Suburu, Cristian A. Strassert, Mathias Schenk, Lilo Greune, Christian Rüter, Petra Dersch, Wolfgang Hartmann, Claudia Rossig, Dario Neri, Carsten Müller-Tidow, Christian Schwöppe, Christoph Schliemann, Cyrus Khandanpour, Georg Lenz, Wolfgang E. Berdel, Sebastian Bäumer

**Affiliations:** 1 Department of Medicine A, Hematology, Oncology and Pneumology, University Hospital of Muenster, Albert-Schweitzer-Campus 1, 48149 Muenster, Germany; 2 European Institute for Molecular Imaging, University of Münster, Waldeyerstr. 15, 48159 Münster, Germany; 3 Institute for Clinical Radiology, University Hospital Münster, Albert-Schweitzer Campus 1, 48149 Münster, Germany; 4 Division of Antibody-Based Immunotherapy, Christian-Albrechts-University, Arnold-Heller-Straße 3, 24105 Kiel, Germany; 5 Department of Pediatric Hematology and Oncology, University Children’s Hospital Muenster, Albert-Schweitzer-Campus 1, 48149 Muenster, Germany; 6 Department of Inorganic and Analytical Chemistry, University of Muenster, Corrensstraße 28/30, 48149 Münster, Germany; 7 Department of Drug Design and Target Validation, Fraunhofer Institute for Cell Therapy and Immunology IZI, Weinbergweg 22, 06120 Halle (Saale), Germany; 8 Institute for Infectiology, Center for Molecular Biology of Inflammation (ZMBE), University of Münster, Von-Esmarch-Str. 56, 48149 Münster, Germany; 9 Gerhard-Domagk Institute for Pathology, University of Muenster, Albert-Schweitzer-Campus 1, 48149 Muenster, Germany; 10 Institute of Pharmaceutical Sciences, ETH Zurich, Vladimir-Prelog-Weg 1-5/10, 8093 Zurich, Switzerland; 11 Department of Medicine V, University of Heidelberg, Im Neuenheimer Feld 410, 69120 Heidelberg, Germany

**Keywords:** RNA interference, Gemtuzumab, DNMT3A inhibition, Ibrutinib, Molecular targeted therapy

## Abstract

**Background:**

Acute myeloid leukemia (AML) is a fatal clonal hematopoietic malignancy, which results from the accumulation of several genetic aberrations in myeloid progenitor cells, with a worldwide 5-year survival prognosis of about 30%. Therefore, the development of more effective therapeutics with novel mode of action is urgently demanded. One common mutated gene in the AML is the DNA-methyltransferase DNMT3A whose function in the development and maintenance of AML is still unclear. To specifically target “undruggable” oncogenes, we initially invented an RNAi-based targeted therapy option that uses the internalization capacity of a colorectal cancer specific anti-EGFR-antibody bound to cationic protamine and the anionic siRNA. Here, we present a new experimental platform technology of molecular oncogene targeting in AML.

**Methods:**

Our AML-targeting system consists of an internalizing anti-CD33-antibody–protamine conjugate, which together with anionic molecules such as siRNA or ibrutinib-Cy3.5 and cationic free protamine spontaneously assembles into vesicular nanocarriers in aqueous solution. These nanocarriers were analyzed concerning their physical properties and relevant characteristics in vitro in cell lines and in vivo in xenograft tumor models and patient-derived xenograft leukemia models with the aim to prepare them for translation into clinical application.

**Results:**

The nanocarriers formed depend on a balanced electrostatic combination of the positively charged cationic protamine-conjugated anti-CD33 antibody, unbound cationic protamine and the anionic cargo. This nanocarrier transports its cargo safely into the AML target cells and has therapeutic activity against AML in vitro and in vivo. siRNAs directed specifically against two common mutated genes in the AML, the DNA-methyltransferase DNMT3A and FLT3-ITD lead to a reduction of clonal growth in vitro in AML cell lines and inhibit tumor growth in vivo in xenotransplanted cell lines. Moreover, oncogene knockdown of DNMT3A leads to increased survival of mice carrying leukemia patient-derived xenografts. Furthermore, an anionic derivative of the approved Bruton’s kinase (BTK) inhibitor ibrutinib, ibrutinib-Cy3.5, is also transported by this nanocarrier into AML cells and decreases colony formation.

**Conclusions:**

We report important results toward innovative personalized, targeted treatment options via electrostatic nanocarrier therapy in AML.

**Supplementary Information:**

The online version contains supplementary material available at 10.1186/s13045-022-01390-5.

## Background

Acute myeloid leukemia (AML) is one of the most prevalent hematological malignancies worldwide [1] with a 5-year survival prognosis of about 30% [2]. Therefore, the development of more effective therapeutics with novel mode of action is urgently demanded. One attempt to achieve this aim is the development of specific molecular therapeutics that target specifically leukemia cells and/or oncogenes. Here, we introduce a modular adjustable antibody–protamine nanocarrier platform, which can electrostatically bind anionic components and transport them via target-cell specific receptor-internalization into leukemia cells. The anionic components can be oncogene-specific small interfering (si)RNAs [3–5] or anionic small molecule inhibitors [6].

The use of RNA interference (RNAi) has been proposed for many cancers, but so far no RNAi-based therapeutic is approved for oncological diseases. RNAi requires the application of specific siRNAs that mediate an intracellular specific mRNA knockdown of any desired target gene [7–9]. Up to now, serum stability and lack of targeted transport of siRNA prohibited their systemic application against cancer. We therefore developed an RNAi-based targeted therapy platform technology that complexes anionic siRNA to cationic nucleic acid-binding protein protamine, which we conjugated chemically to a cancer-cell specific antibody. To this end, we have previously obtained a stable complex comprised of the internalizing anti-EGFR-antibody cetuximab bound to cationic protamine and the anionic siRNA against KRAS and PIK3CA that specifically enters colorectal cancer cells [3–5]. Parallel to this study, we observed that these complexes form suprastructures that resemble micelles and act as nanocarriers [10]. As a modular part of our nanocarrier system, siRNA can be designed and synthetized against any oncogenic factor and we here propose a targeted siRNA-transport system composed of an anti-CD33-antibody, protamine and siRNA that efficiently targets the leukemia-related oncogenes DNMT3A and FLT3.

It is widely accepted that DNA methylation is an important regulatory mechanism in the development and maintenance of AML. DNMT3A is a de novo DNA methyltransferase that has attracted much attention because of its frequent mutation in a large variety of hematologic neoplasms [11]. Moreover, DNMT3A mutations precede cancer development in clonal hematopoiesis and are believed to induce methylation changes of tumor suppressor genes in AML. Mutations in DNMT3A are associated with a poor prognosis [12, 13]. The most common mutation in the *DNMT3A* gene, R882H, has a dominant negative activity that reduces DNA methylation activity [14]. This indicates that DNMT3A has a role in the induction and maintenance of AML and, consequently, is an interesting target in the treatment of DNMT3A-mutated AML. DNMT3A-mutations usually coexist with other AML-oncogenes, 60–80% with NPM1c and 35–41% with FLT3-ITD [12, 15–17]. An effective molecular targeted therapy could include the simultaneous inhibition of, e.g., FLT3-ITD and DNMT3A, of which DNMT3A so far is undruggable.

Our novel approach is the assembly of an antibody-based siRNA nanocarrier to induce specific RNAi against mutated DNMT3A and FLT3 in leukemia. As a candidate surface molecule for a preferential delivery into leukemic cells, CD33 is expressed on blasts in almost all AML patients, sparing hematopoietic stem cells, lymphoid cells and non-hematopoietic cells [18, 19]. The anti-CD33-antibody we use is gemtuzumab, which is known as the internalizing part of gemtuzumab-ozogamicin (Mylotarg®, GO), an antibody–drug conjugate approved for AML in a combination therapy with chemotherapy [20]. GO is an antibody-targeted chemotherapeutic drug consisting of the humanized murine CD33 antibody (clone P67.6, gemtuzumab) to which the calicheamicin derivative ozogamicin is attached via a hydrolyzable linker. It is assumed that binding of GO to the CD33 antigen results in internalization followed by the release of the potent antitumor antibiotic ozogamicin. GO was first approved by the FDA in 2000, withdrawn in 2010 and later re-approved for AML in 2017, but comes with a box warning for hepatotoxicity, which is most likely related to complex bystander-effects of ozogamicin [21], but not the drug carrier antibody P67.6/gemtuzumab, since the latter had no effect on the cells, if applied unconjugated [22].

Using these novel antibody-targeted nanocarriers for siRNA, we achieved downregulation of DNMT3A and FLT3 that lead to significantly decreased cell viability and reduced clonal colony growth of leukemic cells in vitro. Further, we observed decreased growth of xenograft-transplanted AML cells as well as prolongation of survival in patient-derived xenotransplant (PDX) mouse model of AML in vivo.

For the targeted treatment of Diffuse-Large B-Cell Lymphoma (DLBCL), we established a targeted chemically modified ibrutinib, ibrutinib-Cy3.5, which was incorporated into a nanocarrier specifically internalizing in CD20-positive cells [6]. Ibrutinib is a covalent binder of BTK, which also has been proposed to be a relevant drug target in AML [23]. To illustrate the platform-characteristic of our anti-CD33-mAB-based nanocarrier, we show that ibrutinib-Cy3.5 can act as an efficient inhibitor of BTK in AML cells, as postulated before [24–26].

Therefore, the development of different AML-specific nanocarriers shows the strength of the electrostatic nanocarrier platform technology that will be applicable to many cell types and target genes of interest.

## Methods

### Cloning and coupling of anti-CD33 monoclonal antibody

To express the humanized monoclonal anti-CD33-antibody (αCD33-mAB) gemtuzumab in a mammalian expression system, pVITRO2-neo-mcs_Gemtuzumab-IRES-GFP plasmid was transfected into CHO-S cells. αCD33-mAB was purified from CHO-S clone supernatants using General Electric Äkta pure chromatography system with a CH1 column and UNICORN software followed by dialysis and storage in PBS at 4 °C for several months.

Protamine sulfate (3.9 mM) (Cat. No. 539122, Calbiochem) was amino-terminally coupled to sulfo-SMCC (Pierce No. 22622, Rockford, IL, USA) at a molar ratio of 1:5 in ddH_2_O (pH adjusted to 6.0–7.0 with 0.1 M carbonate buffer (pH 8.3)) and incubated for 1 h at 37 °C, purified by gel filtration chromatography in Zeba spin desalting columns (Pierce No. 89891). Resulting protamine-sulfo-SMCC was coupled to cysteine residues of αCD33-mAB in a 32:1 or 120:1 molar ratio at 4 °C overnight, unless stated otherwise. After sterile filtration, the αCD33-mAB-protamine (αCD33-mAB-P/P) complex was stored at 4 °C and was stable for several weeks.

### Depletion of free protamine from the αCD33-mAB-protamine complex

After chemical conjugation, αCD33-mAB-P containing unbound SMCC-protamine in molecular excess was applied to protein G-sepharose equilibrated with PBS, washed with 10 CV of PBS and then eluted with a steep gradient of 100 mM glycine–HCl pH 2.5. Fractions were collected and checked for presence of unbound SMCC-protamine by SDS–PAGE and Coomassie stain. Fractions depleted of unbound SMCC-protamine were subjected to further analysis.

### siRNA—anti-CD33-mAB-protamine coupling

The coupling of 5–20-fold molar excess siRNA duplexes to αCD33-mAB-P/P (anti-CD33-mAB-protamine/free protamine) was performed for 2 h at room temperature (RT) freshly before use. For valuation of siRNA binding and internalization efficiency, αCD33-mAB-P/P was coupled to Allstar negative control siRNA-Alexa488 (cat. No. 1027284, Qiagen, Hilden, Germany). Treatment experiments were done using siRNA duplexes against wild type and mutant DNMT3A (sense: 5’ GAACAGAAGGAGACCAACA-dAdA), a 1:1 mixture of two FLT3 siRNAs acting against wild type and mutant FLT3 (siRNA1 sense: 5’-GAA UUU AAG UCG UGU GUU CUU-rUrU and siRNA2 sense: 5’-CGC AAC AGC UUA UGG AAU UUU-rUrU) and a scrambled siRNA (sense: 5’ GGC CAG ACA CCG UCA UUU AA-dTdT; all Dharmacon, Colorado, USA) as control.

### Cell culture

Human AML cell lines KG1 (ACC 14; KRAS-G12D), OCI-AML2 (ACC 99; homozygous DNMT3A-R635W), MV4-11 (FLT3-ITD/MLL-AF4) and OCI-AML3 (ACC 582; DNMT3A-R882C, NPM1c-mut, homozygous NRAS-Q71L) and the DLBCL cell line OCI-Ly19 (NRAS-Q61K; all DSMZ, Braunschweig, Germany) were maintained in RPMI-1640 medium, supplemented with 10% fetal bovine serum (FBS) and 1% streptomycin and penicillin at cell densities of 0.2 – 1 × 10^6^ cells/ml and incubated at 37 °C with 5% CO_2_ and high humidity. The EGFR-positive NSCLC cell line A549 was treated with antibody-siRNA-complexes as described previously [10] and cultivated in DMEM supplemented with 10% FBS and 1% penicillin and streptomycin.

### Flow cytometry

For detection of the CD33-molecule, AML cells were incubated with 4 µl mouse anti-human CD33 (cat. No. 555625, BD Biosciences) in 100 µl PBS for 30 min at 4 °C. Next, cells were washed 3 times with PBS and stained with 2 µl PE-labeled goat anti-mouse IgG (cat. No. 550589, BD Biosciences) in 100 µl PBS for 30 min at 4 °C. Cells were washed again with PBS and subjected to flow cytometric analysis.

For verification of antibody internalization, 2 × 10^5^ cells were washed with PBS and incubated with 30 nM αCD33-mAB-P/P for 1 h at 37 °C. After three times washing with PBS, cells were treated with purified mouse anti-human CD33 and stained with PE-labeled goat anti-mouse IgG as described above. Internalization was determined via flow cytometry.

### Detection of internalized vesicles in cells and vesicular nanocarrier by fluorescence microscopy

For detection of internalized vesicles in cells, 60 nM αCD33-mAB-P/P complex was coupled to 600 nM Allstar negative control siRNA-Alexa488 for 2 h at RT. 5 × 10^4^ cells were seeded and treated with this nanocarrier over night at 37 °C and 5% CO_2_. Subsequently, cells were washed with cold PBS, transferred to adhesion slides bordered with liquid pen and kept on ice, incubated for 20 min to adhere to surface, fixed with ice-cold 4% paraformaldehyde (PFA), stained with Hoechst33342, mounted with DAKO fluorescent mounting medium (lot. no. 10121691, DAKO, North America), covered with cover slips and photographed on a Nikon Eclipse 50i upright microscope.

### Analysis of nanocarrier formation by fluorescence microscopy

For cell-free nanoparticle-self-assembly studies, preformed αCD33-mAB-P/P-Alexa488-siRNA nanocarriers were applied to cell-culture treated chamber slides overnight (o/n) in order to settle the nanoparticles by gravity, washed with PBS, fixed with 4% PFA and mounted in DAKO mounting medium for microscopical analysis. Nanocarrier stability was tested by incubation of preformed nanocarriers for 24 h on chamber slides in buffers with the indicated pH values (pH 4–8) or for 24–72 h with PBS, PBS/10% FCS or PBS/50% FCS, respectively. Slides were washed, fixed and analyzed as described above.

For the detection of the antibody position on the nanocarriers, these nanocarriers were stained with Alexa647-anti-hIgG (Dianova, Germany, #109–607-003) after fixation and mounted in DAKO mounting medium for microscopical analysis.

Protamine was conjugated using Cy3 Mono-Reactive Dye Pack (cat. no. PA23001, Cytiva, Freiburg, Germany) according to the manufacturer’s protocol to achieve protamine-Cy3.

### Western blot analysis

αCD33-mAB-P/P was coupled to control-siRNA or DNMT3A- or FLT3-siRNA in a 1:5 to 1:20 molar ratio as indicated for 2 h at RT. 5 × 10^5^ cells of each cell line were seeded and treated with the αCD33-mAB-P/P-siRNA nanocarriers daily for 3 days.

Isolated tumors were admitted in tenfold amount of RIPA Lysis buffer, reduced with Ultra Turax and cleared via ultrasonification and centrifugation.

Western blot analysis was performed using standard protocols with the following antibodies: anti-DNMT3A (D23G1, rabbit mAB, Cell Signaling), anti-FLT3 (S-18, rabbit pAb, Santa Cruz), anti-ß-actin (A5441, mouse mAB, Sigma) and anti LaminB1 (cat. No. 12987–1-AP, Proteintech).

### CellTiter-Glo assay

Cells viability was assessed using CellTiter-Glo luminescent cell viability assay system according to the manufacturer’s recommendations (cat. No. G7571, Promega, Madison, WI, USA).

### Colony forming assays

200 cells of each cell line were treated with the indicated αCD33-mAB-P/P-siRNA nanocarrier for 2 h at 37 °C and seeded in methylcellulose (cat. No. 03231, Stemcell Technologies, Vancouver) in 96-wells (OCI-AML2, OCI-Ly19 and KG1) or 48-wells (OCI-AML3 and MV4-11) for 5–7 days. Colony formation in soft agar for A549 cells was performed as described previously [10].

The study was reviewed and approved by the ethics committee of the Physician´s Chamber of Westphalia-Lippe and the medical faculty of the University of Muenster (2007–524-f-S and 2007–390-f-S) before the study began. AML samples were obtained from bone marrow of patients with acute myeloid leukemia at the time of initial diagnosis. The median blast count was 80%. CD34^+^/CD33^+^ cells from frozen bone marrow cells of AML patients, or CD34^+^/CD19^−^ peripheral blood mononuclear cells (PNMCs) from healthy persons (n = 3), respectively, were treated at equal cell numbers with αCD33-mAB-P/P-siRNA and seeded in human methylcellulose (R&D Systems, Minneapolis, USA). After 1 – 2 weeks the assay was stained with 1 mg/ml INT (Iodonitrotetrazolium chloride), incubated over night at 37 °C and colonies were counted via binocular.

### Dynamic light scattering (DLS)

Particle size detection by means of dynamic light scattering (DLS) was performed on a zeta-counter (MALVERN, Malvern, United Kingdom), which correlates light diffusion caused by particles in a solution to their size and the zeta-potential. Measurements were performed in at least three technical replicates.

### Electron microscopy

Freshly prepared nanoparticles were sedimented on a formvar-coated, carbon-sputtered copper grid. After negative staining with 1% phosphotungstic acid, pH 7.0, the samples were analyzed at 80 kV on a Tecnai 12 electron microscope (Fei, Eindhoven, The Netherlands). Images of selected areas were documented with Veleta 4 k CCD camera (Emsis, Münster, Germany).

### In vitro assay for toll-like receptor (TLR) activation

THP1-Dual™ (InvivoGen, Toulouse, France) cells were cultivated as recommended and treated with nanoparticles loaded with indicated effective vs control siRNAs, as well as indicated nanocarriers loaded with ibrutinib for 24 h at cell-culture conditions (60 nM antibody or nanocarrier, respectively, 300 nM free or complexed siRNA, 1200 nM free or complexed ibrutinib + -Cy3.5). Results were controlled by uncomplexed siRNAs and PBS treatment. Soluble luciferase LUCIA and SEAP levels were determined on a Victor plate reader (PerkinElmer, Rodgau, Germany) according to the manufacturer’s recommendations using QUANTI-Luc and QUANTI-Blue substrates (InvivoGen, Toulouse, France).

### Mouse xenograft tumor models

All animal experiments in this study were carried out in accordance with the recommendations of the Institutional Animal Care and Use Committee “Landesamt für Natur, Umwelt und Verbraucherschutz NRW” (LANUV). This study was performed with permission of the Institutional Animal Care and Use Committee and of the local veterinary administration of Muenster (Permit Numbers 84–02.04.2014.A193 and 81–02.04.2019.A341).

1 × 10^7^ OCI-AML2, KG1 or MV4-11 cells, respectively, were transplanted subcutaneously into 10–15 week-old female CD1 nude mice. After tumors reached a size of 150 – 300 mm^3^, mice were randomized into treatment groups. Mice were treated 3 times weekly intraperitoneally with the indicated agents such as PBS, αCD33-mAB-P/P-scr-siRNA (scr, scrambled), αCD33-mAB-P/P-DNMT3A-siRNA or αCD33-mAB-P/P-FLT3-siRNA, respectively, at 2 mg/kg referred to the αCD33-mAB-P/P with a 10 times molar excess of siRNA. Tumor growth was measured with caliper and tumor volume was calculated by the formula length x width^2^ × 0.52. Animals were euthanized and prepared for further analysis including analysis of serum parameters, when the first mouse reached a tumor volume of approx. 1500 mm^3^. Serum was analyzed for blood urea nitrogen (BUN), serum creatinine (CR), and the liver enzyme AST (aspartate aminotransferase; other name: glutamic oxaloacetic transaminase (GOT)) and ALT (alanine aminotransferase; other name: glutamic pyruvic transaminase (GPT)) values in a standard clinical hematological laboratory setting. Thus, absolute standard values often found for CD1 mice are not applicable. For immunostaining, isolated tumors were washed in PBS, fixed in 4% paraformaldehyde (PFA) in PBS and embedded in cryomatrix. For detection of apoptotic cells, TUNEL Assay Kit—HRP-DAB (Abcam cat. no. ab206386) was used according to the manufacturer’s recommendations. For Ki67 and DNMT3A staining, dried cryomatrix sections were blocked in blocking solution (2% normal horse serum, 0.1% Tween 20 in TBS), washed three times in TBS/0.1% Tween 20 and incubated with Ki67 antibody (rabbit mAB clone D2H10) or DNMT3A antibody (DNMT3A D23G1 Rabbit mAB #3598) diluted 1:300 in blocking solution overnight at 4 °C. After sections were washed three times in TBS/0.1% Tween 20 for 5 min, secondary antibodies (goat anti-rabbit-Alexa488 diluted 1:10,000 in blocking solution) were applied for 1–2 h at RT. Counterstaining was performed using Hoechst 33,342.

### Quantitative real-time PCR (qPCR) of xenograft tumors

RNA and cDNA were prepared from tumors and expression analysis performed using standard procedures with the following probes: DNMT3A (Hs01027166), HoxA11 (Hs00194149), Meis1 (Hs01017441), Bcl2 (Hs00236329), MYC (Hs00153408, all Thermo Fisher) with ABL as control.

### RNAseq analysis

Library preparation of total RNA from OCI-AML2 xenotransplanted tumors was performed with the NEBNext Ultra II RNA directional Kit and single read sequencing was performed using a NextSeq® 2000 System with a read length of 72 bp. Using a molecular barcode, the samples were demultiplexed (bcl2fastq2) to fastq data and quality controlled (FastQC). Trimmomatic was used for adapter trimming and read filtering1. The resulting reads were aligned to the Ensembl GRCh38 reference genome using Hisat22. The aligned reads were sorted using samtools3. The sorted and aligned reads were counted into genes using htsec-counts [27]. The test for differential expression was performed using the r-package deseq2 [28]. Data are available on request from the authors.

The GSEA analysis was done using GSEA software version 4.1.0 [29], which uses predefined gene sets from the Molecular Signatures Database (MSigDB v7.4). A gene set is a group of genes that shares pathways and functions. For the present study, we used hallmark signatures for GSEA analysis. The minimum and maximum criteria for selection of gene sets from the collection were 10 and 500 genes, respectively. Different combinations were tested: PBS VS DM3A, SCR VS DM3A, PBS + SCR VS DM3A and PBS VS SCR. False detection rate of 5% and a nominal p value of *p* < 0.05 was used to filter the gene signatures. The most significant hall mark signature pathways are presented. To better understand our RNA sequencing data and GSEA observations, genes contributing to core enrichment were further analyzed to characterize potential protein–protein interactions (PPIs) using STRING database (Version 11.5 [30, 31]). PPIs with a confidence score ≥ 0.4 were filtered and subsequently analyzed via cytoHubba plug-in [32] of cytoscape (Ver 3.9.1 [33, 34]) to identify important hub genes. cytoHubba employs 12 different algorithms to identify potential PPIs reflecting contribution of the hub genes in a PPI network from different aspects. Briefly, we analyzed our data (Hallmark_oxidative phosphorylation and c-MYC targets V1 gene sets) as previously described [35]. PPIs were analyzed using String/Cytoscape/cytoHubba interface and queried for top 15 hub genes using degree-based algorithm and presented as a circular layout and ranked based on scores. Complete PPI network/subnetworks were analyzed using additional 11 algorithms for top 15 hub genes for each method. Hub genes representing intersection of at least nine methods (75% overlapping signature) that indicate a promising PPI network were presented.

### PDX mouse model

AML blasts from patient #751 were primary transplanted intravenously in two NSG mice (Charles River, Germany). Blood was tested for human CD33^+^/CD45^+^ cells via flow cytometry analysis every two weeks. When percentage of AML blasts reached 40%, mice were euthanized, bone marrow and spleen were dissected and cells were isolated and stored in liquid nitrogen for further use. For the treatment experiment, 1.1 × 10^6^ bone marrow and spleen cells were secondarily transplanted intravenously into 20 NSG mice. Mouse blood was tested for human CD45^+^ cells every week. When the percentage of human AML cells reached a mean of 6%, mice were randomized into treatment groups. Mice were treated three times per week intraperitoneally with PBS, αCD33-mAB-P/P-scr-siRNA, αCD33-mAB-P/P-DNMT3A-siRNA or αCD33-mAB-P/P-FLT3-siRNA, respectively, at 4 mg/kg referred to the αCD33-mAB-P with a 5 times molar excess of siRNA. As progress control, blood from some mice was repeatedly tested for human CD33^+^/CD45^+^ cells via flow cytometry. When mice showed first signs of illness, they were euthanized and dissected as described below. The life span from start of treatment to death was statistically evaluated in a Kaplan–Meier survival curve.

### Biodistribution studies

For preliminary biodistribution studies, mice carrying either subcutaneous OCI-AML2 tumors or engrafted PDX cells as described above were treated with nanocarriers complexed with Cy5-DNMT3A-siRNA (sequence as above) or non-labeled control by i.p. injection. After 24 h, mice (n = 2 in each group) were sacrificed, organs and/or tumors were excised and placed at a petri dish and imaged in a Fluorescence Reflectance Imager (FRI, in vivo MS FX Pro, Bruker Biospin MRI GmbH, Ettlingen, Germany). First, a white light image was taken, followed by a fluorescence image with an exposure time of 30 s. The fluorescent tag was excited with light at a wavelength of 630 nm (± 10 nm) and fluorescence was registered within the wavelength of 700 nm (± 17.5 nm). The data were analyzed with the imaging software MISE (Bruker, v 7.5.2). After *ex vivo* imaging, organs were processed for cryosectioning according to standard protocols, sectioned, mounted on Superfrost-slides and processed for fluorescence microscopy for Cy5 signals. In parallel, adjacent sections were fixed in PBS/4% PFA and immunolabeled with anti-human IgG-488 (Dianova #109–547-003) diluted 1:200 in PBS for 2 h at RT, washed and mounted for immunofluorescence microscopy as described above.

### Ibrutinib-Cy3.5 and αCD33-mAB-P/P complex formation

Anionic charged ibrutinib-Cy3.5 was synthesized as published recently [6] and complexed in 20 times molar excess to αCD33-mAB-P/P, if not stated otherwise, for ibrutinib at room temperature in the dark for 30–60 min. Complexes were prepared freshly before use.

### Determination of ibrutinib-Cy3.5 load capacity, nanoparticle formation and functional analysis

For the analysis of in vitro-formation of nanoparticles, 1200 nM ibrutinib-Cy3.5 was complexed in 60 nM αCD33-mAB-P/P and subjected to gel mobility shift assays, microscopic analysis and Western blots essentially as described [6] and in analogy to the methods described above using siRNA.

For BTK occupancy analysis, OCI-AML2 cells were treated with PBS, 1200 nM ibrutinib-Bodipy-FL (PCI-33380, cat. no. HY-100335, Hölzel-Diagnostika, Cologne, Germany) or 1200 nM ibrutinib for 4 h, washed with medium and treated o/n with 60 nM αCD33-mAB-P/P complexed with 1200 nM ibrutinib-Cy3.5 and subjected to fluorescence microscopy as described above. Colony formation assays using αCD33-mAB-P/P-ibrutinib-Cy3.5 was performed essentially as described above for OCI-AML2 cells using 1200 nM ibrutinib-Cy3.5 or uncharged ibrutinib or 60 nM αCD33-mAB-P/P complexed with 1200 nM ibrutinib-Cy3.5.

### Statistical analysis

All data are presented as means ± or + standard deviation (SD), if not indicated otherwise. All p-values are representing two-tailed T-test analysis if not stated otherwise. P-values < 0.05 were accepted as indicating significant differences.

## Results

### Development of an AML-specific siRNA carrier system

**Fig. 1:**
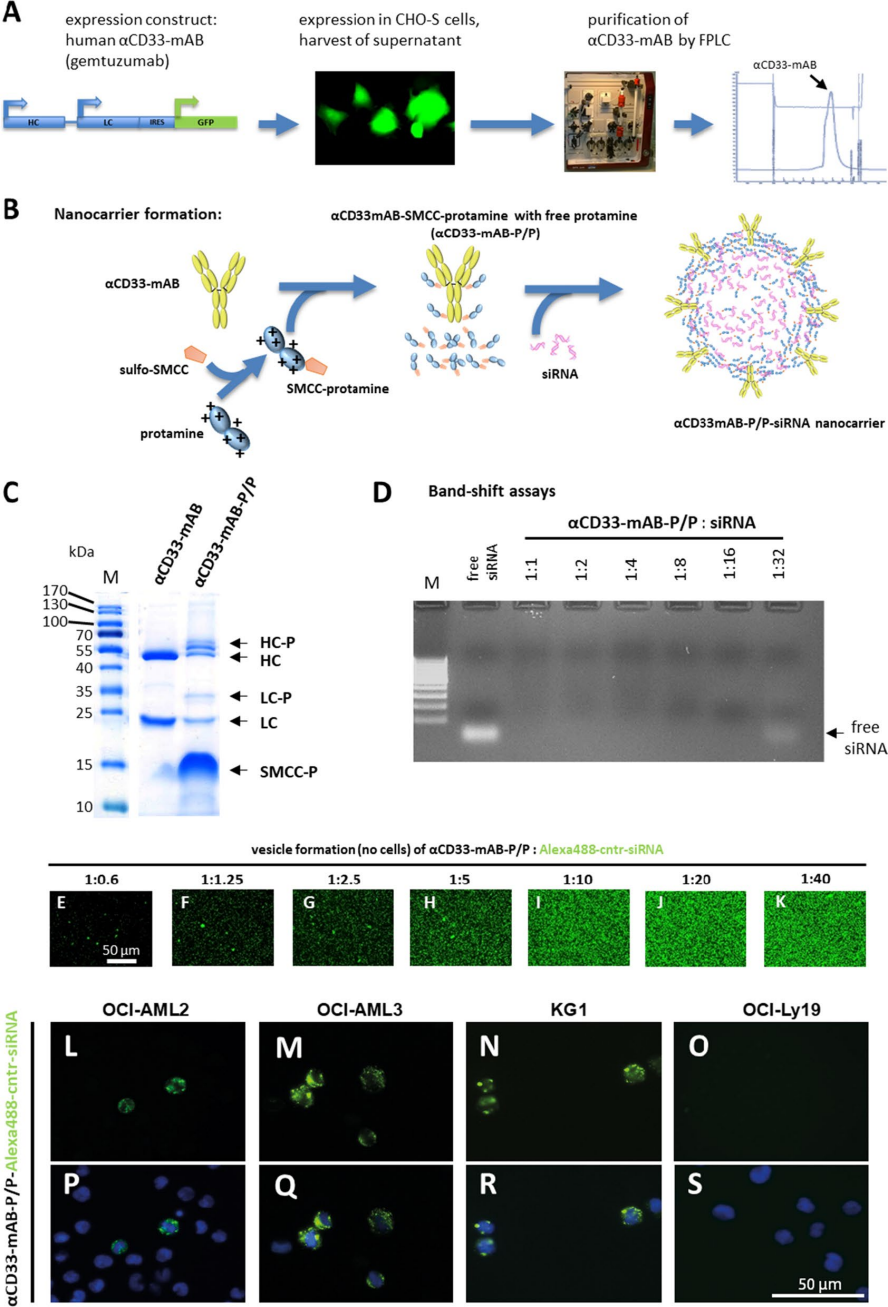
The αCD33-monoclonal antibody (mAB) gemtuzumab-protamine (αCD33-mAB-P/P) conjugates bind and transport siRNA only into CD33-positive cells. **A** We cloned a plasmid expressing the heavy (HC) and light chain (LC) of the full IgG1 monoclonal antibody gemtuzumab. After transfection of the production cell line CHO-S, the mAB was purified via FPLC. Shown here is a representative elution profile (right panel). IRES, internal ribosomal entry side; GFP, green fluorescent protein. **B** First, protamine was conjugated chemically to the linker sulfo-SMCC, then this linker was coupled to the αCD33-mAB. The naturally anionic siRNA could then complex by electrostatic binding to the protamine moieties. In complex with siRNA, the αCD33-mAB-P and free SMCC-protamine spontaneously form vesicular structures, which we call αCD33-mAB-P/P-siRNA nanocarrier. **C** Coomassie-stained SDS–PAGE showing uncoupled αCD33-mAB and αCD33-mAB coupled with SMCC-protamine (αCD33-mAB-P/P). The shift of molecular weight after protamine conjugation via sulfo-SMCC to the αCD33-mAB can be seen for the heavy chain (HC-P) and for the light chain (LC-P). SMCC-P, unbound SMCC-protamine; M, molecular weight marker. **D** Band-shift assay. Agarose gel-electrophoretic analysis of the binding capacity of siRNA to the αCD33-mAB-P/P complex. Up to 16 mol siRNA per mol of mAB-P can bind to the αCD33-mAB-P/P complex. **E–K** Fluorescence microscopy of nanocarriers. Vesicles were formed by self-assembly upon incubation of 60 nM αCD33-mAB-P/P with 600 nM Alexa488-control-siRNA for 2 h at RT and documented by fluorescence microscopy after immobilization of the nanocarriers on coated slides without cells o/n at 37 °C. **L–S** Fluorescence microscopy analysis of siRNA internalization mediated by αCD33-mAB-P/P-siRNA complex into CD33-positive cells (L-N and P-R) but not into negative control cells (O and S). Upper panels (L-O): Green fluorescence depicting Alexa488-control siRNA in vesicular structures within the cells. Lower panels (P-S): Overlay of blue Hoechst staining and green fluorescence from upper panels. α, anti

In order to develop an siRNA-based targeted therapy to treat leukemia, we chose the humanized anti-CD33-monoclonal antibody (αCD33-mAB) gemtuzumab as a carrier. We expressed the αCD33-mAB in CHO-S-cells (Fig. 1A) and isolated it via affinity chromatography (Fig. 1A and B). Then, the αCD33-mAB was conjugated via the linker sulfo-SMCC to commercially available salmon protamine (Fig. 1B). As depicted here, we found out that antibodies coupled with SMCC-protamine form vesicular structures in the presence of free SMCC-protamine when they were complexed with anionic cargo molecules such as siRNA (see below in this manuscript and [10]) or anionic ibrutinib-Cy3.5 [6]. Therefore, we abbreviate the empty carrier components as αCD33-mAB-P/P. Cysteine residues within the αCD33-mAB were conjugated to SMCC-protamine, detectable by a molecular weight shift (Fig. 1B), which resulted in the antibody–protamine complex (Fig. 1C). As revealed by electrophoretic band-shift assays, the αCD33-mAB-P/P can complex around 16 molecules of siRNA, while a higher molar excess of siRNA leads to an overflow of unbound siRNA (Fig. 1D). Specific CD33 internalization induced by αCD33-mAB-protamine complex incubation of CD33-positive cells was confirmed by flow cytometry (Additional file 1: Figure S1 A-D). Moreover, αCD33-mAB-P/P-siRNA complexes internalized efficiently and specifically into CD33-positive OCI-AML2, OCI-AML3 and KG1 AML cells (Fig. 1L-N and P-R), whereas CD33-negative OCI-Ly19 DLBCL cells did not internalize αCD33-mAB-P/P-Alexa488-control-siRNA complexes (Fig. 1O and S). As anticipated when αCD33-mAB-P/P-Alexa488-control-siRNA complexes were incubated on coated slides overnight, micellar-like Alexa488-positive structures appeared (Fig. 1E-K) depending on the αCD33-mAB-P/P:siRNA ratio, illustrating the formation of a supramolecular complex that we call αCD33-mAB-P/P-siRNA nanocarrier.

### The αCD33-mAB-P/P-nanocarrier directed RNAi inhibits target gene expression and colony growth in DNMT3A-mutated CD33-expressing AML cell lines

**Fig. 2:**
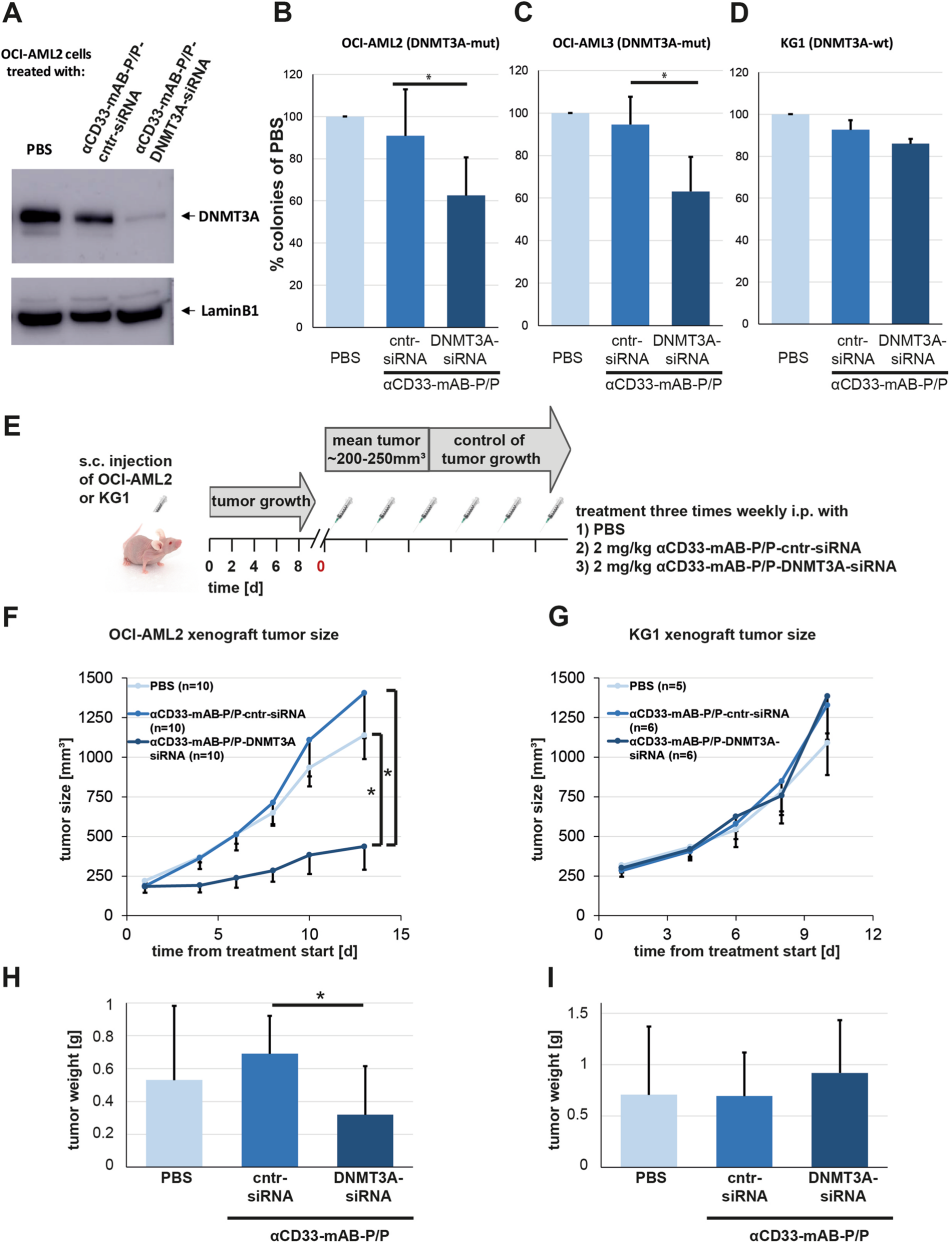
αCD33-mAB-P/P-siRNA-mediated RNAi inhibits target gene expression and decreased growth of DNMT3A-mutant AML cells in vitro and in vivo. **A** Western blot analysis of treated OCI-AML2 cells. **B–D** Colony formation assay of DNMT3A mutant cell lines OCI-AML2 (**B**, n = 6) OCI-AML3 (**C**, n = 6) and DNMT3A-wild type KG1 cells (**D**, n = 3). There is a significant decrease in colony growth due to αCD33-mAB-P/P-DNMT3A-siRNA treatment in OCI-AML2 and OCI-AML3 (B-C, in contrast to control-siRNA), but not in KG1 (D). Significance: *, *p* < 0.05, 2-tailed T-test. Means plus SD of three independent experiments. **E.** Schematic overview about in vivo treatments after subcutaneous (s.c.) injection of 1 × 10^7^ AML cells in CD1-nude mice. Mice were treated intraperitoneally (i.p.) with PBS, αCD33-mAB-P/P-control (cntr)-siRNA or αCD33-mAB-P/P-DNMT3A-siRNA three times weekly. **F,G** Tumor growth curves of OCI-AML2 (F) and KG1 (G). Growth of OCI-AML2 tumors was significantly delayed due to systemic αCD33-mAB-P/P-DNMT3A-siRNA treatment in contrast to control-siRNA treatment and PBS treatment (G), whereas KG1 tumors (DNMT3A wild type) did not show significant differences in tumor growth (G). **H,I** Tumor weight of isolated OCI-AML2 (H) and KG1 (I) tumors. Shown are means plus SD: T-test *, *p* < 0.05. α, anti

The DNA-methyltransferase DNMT3A is mutated in a high number of AML patients (20–30%, [12, 15, 16]), however, the consequences of this mutation event are not fully understood today. According to its prominent abundance even preceding leukemogenesis, we hypothesized an oncogenic function of DNMT3A mutated protein driving AML and intended to inhibit it via RNAi. We verified by Western blot analysis that treatment with αCD33-mAB-P/P-DNMT3A-siRNA led to a significant knockdown of DNMT3A in DNMT3A-mutant OCI-AML2 cells (Fig. 2A). Moreover, we observed that the colony forming capacity of DNMT3A-mutant OCI-AML2 (Fig. 2B) and OCI-AML3 (Fig. 2C) cells was significantly decreased, when treated with αCD33-mAB-P/P-DNMT3A-siRNA compared to αCD33-mAB-P/P-scrambled control-siRNA and PBS treatment, while colony formation in DNMT3A-wild type KG1 cells was unchanged (Fig. 2D). In order to control the cell surface molecule specificity of the αCD33-mAB-P/P carrier antibody for the nanocarrier efficacy, we applied αCD33-mAB-P/P-siRNA nanocarriers to cell lines that do not express CD33, for instance A549 non-small cell lung cancer (NSCLC) cells as well as OCI-Ly19 lymphoma cells (Additional file 1: Figure S1 E–F). In KRAS-dependent A549 cells, KRAS-specific siRNA was effective in colony reduction when the respective nanocarrier was decorated with αEGFR mAB cetuximab, but ineffective when decorated with αCD33-mAB gemtuzumab (Additional file 1: Figure S1 E). In CD33-negative, CD20-positive and DNMT3A-independent OCI-Ly19 cells, αCD33-mAB-P/P-decorated DNMT3A nanocarriers had no effect on colony formation (Additional file 1: Figure S1 F). Vice versa, we applied αCD20-mAB-P/P-decorated DNMT3A-siRNA nanocarrier on CD20-negative and DNMT3A-dependent OCI-AML2 cells, which again exhibited no significant effect on colony formation (Additional file 1: Figure S1 G). Moreover, cell proliferation was compromised after DNMT3A-knockdown in OCI-AML2 and OCI-AML3 cells, while KG1 cells were unaffected (data not shown). Hence, we conclude that the efficacy of the αCD33-mAB-P/P-DNMT3A-nanocarrier is indeed dependent on the presence of both, the expression of CD33 on the cell surface and the addiction of the respective cell to the action of the mutated DNA methylase DNMT3A.

### DNMT3A-mutant xenograft tumors are sensitive to DNMT3A-knockdown via αCD33-mAB-P/P-DNMT3A-siRNA treatment

We then performed in vivo evaluation of anti-CD33-mAB-P/P-siRNA delivery in the DNMT3A-dependent cellular system OCI-AML2 cells in a CD1 nude mouse xenograft model (see Fig. 2E for schematic overview). Besides the systemic transplantation of leukemia cells, the ectopic subcutaneous transplantation of AML cells in immunodeprived mice has a long history in preclinical tests and is widely accepted as a screening experiment for drug evaluation [36–38]. The subcutaneous tumor growth was significantly reduced (*p* < 0.01) upon αCD33-mAB-P/P-DNMT3A-siRNA application, but not upon application of carriers with control siRNA or PBS (Fig. 2F).

Moreover, the knockdown of DNMT3A in wild type KG1 subcutaneous tumors was therapeutically ineffective (Fig. 2G). These results were substantiated by ex vivo tumor weight differences in each treatment cohort (Fig. 2H and I).

**Fig. 3:**
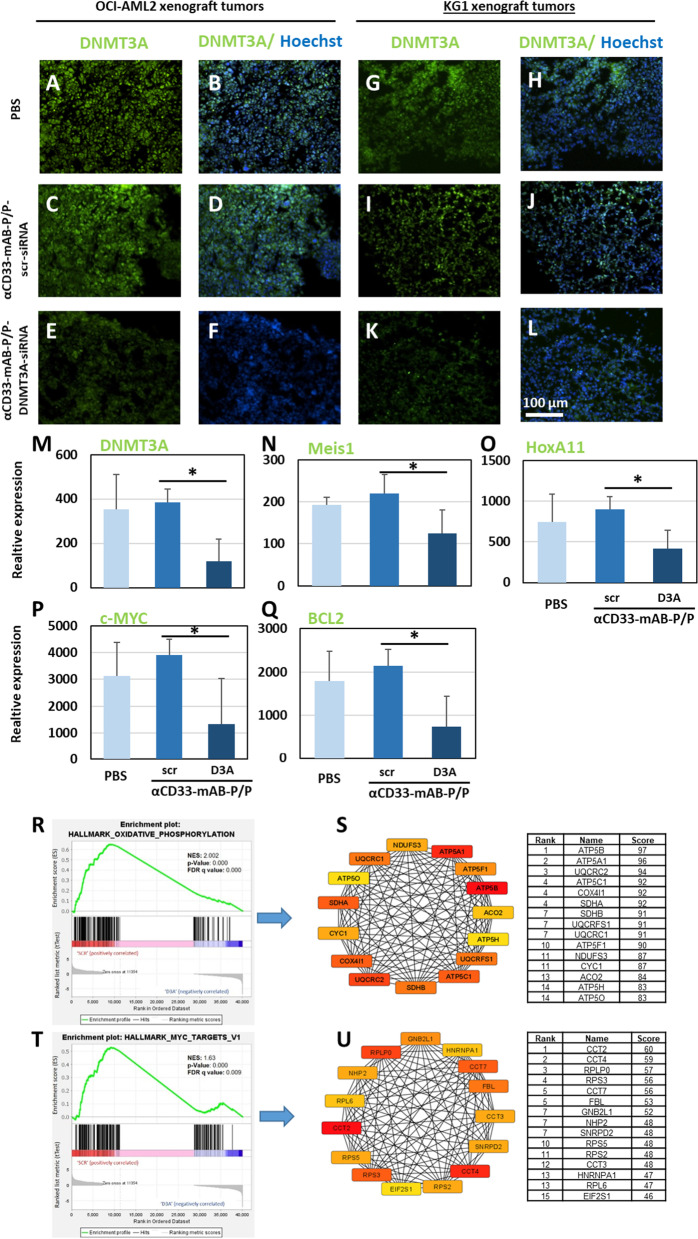
Expression analysis and downstream factors in xenograft tumor samples. **A–L** DNMT3A staining of OCI-AML2 and KG1 tumor sections. DNMT3A was detected in nuclei of OCI-AML2 tumor sections treated with PBS and αCD33-mAB-P/P-control-siRNA; scr, scrambled (= control) (**A–D**), but DNMT3A staining was almost completely lost after treatment with αCD33-mAB-P/P-DNMT3A-siRNA (**E–F**). Reduced DNMT3A staining was also observed in KG1 tumor sections from the αCD33-mAB-P/P-DNMT3A-siRNA treatment group (**K–L** compared to **G–J**). Nuclear counterstain was performed using Hoechst33342 (B, D, F, H, J, L). **M–Q** Relative expression (RT-PCR) of DNMT3A and downstream genes in OCI-AML2 tumors ex vivo upon previous in vivo exposure to PBS, control carriers (αCD33-mAB-P/P-scr-siRNA) and αCD33-mAB-P/P-DNMT3A-siRNA. D3A, DNMT3A siRNA; α, anti. **R,S** Gene set enrichment analysis (GSEA) of RNA sequencing data in αCD33-mAB-P/P-DNMT3A-siRNA vs. scr-siRNA carrier-treated OCI-AML2 tumors, ex vivo*.* GSEA analysis of hallmark gene sets from the molecular signature database identified significant enrichment for hallmark_oxidative phosphorylation **(R)** and hallmark_c-Myc targets **(T)** in αCD33-mAB-P/P-scr-siRNA carrier-treated OCI-AML2 tumors. Shown are representative gene set enrichment plots. FDR, false discovery rate q-value and NES, normalized enrichment score. **S, U** Graphical presentation of central genes within the biological networks identified in the GSEA analysis. Top 15 hub genes identified by String/cytoscape/cytoHubba interface were presented as a circular layout (color coded: red and yellow indicating a higher and lower rank, respectively) using degree based algorithm of cytoHubba plug-in. Rank, gene and the scores were presented in the adjacent table. Furthermore, protein–protein interaction network/subnetworks were analyzed using 12 different algorithms of cytoHubba plug-in and hub genes representing intersection of at least nine algorithms (75% overlapping signature) potentially indicate key DNMT3A related hub genes in inducing c-Myc targets and oxidative phosphorylation. Detailed information on these hub genes and their importance in different cancers and therapy outcome is depicted in Additional file 1: Figure S4

Immunohistochemical analysis of OCI-AML2 xenograft tumors at the end of observation revealed diminished DNMT3A-expression (Fig. 3E–F, compared to A-D), decreased numbers of proliferating Ki67-positive cells (Additional file 1: Figure S2 E–F, compared to A-D) and increased numbers of apoptotic TUNEL-stain positive cells (Additional file 1: Figure S2 Q-R, compared to M–N and O-P) of αCD33-mAB-P/P-DNMT3A-siRNA treated mice in comparison to PBS and to αCD33-mAB-P/P-scr (control)-siRNA treated mice. DNMT3A-expression was also decreased in KG1 xenograft tumors of αCD33-mAB-P/P-DNMT3A-siRNA treated mice (Fig. 3K-L, compared to G-J), this knockdown did not lead to decreased Ki67 (Additional file 1: Figure S2 G-L) or increased TUNEL positive cells (not shown). These results underline that specifically DNMT3A-mutated cells, but not DNMT3A-wild type cells were vulnerable to DNMT3A-knockdown in vivo.

To get a deeper insight in downstream effects of DNMT3A-knockdown in vivo, we further analyzed RNA from OCI-AML2 tumors samples. Real-Time RT-PCR for different formerly identified downstream target factors of DNMT3A function revealed – besides the significant downregulation of DNMT3A itself (Fig. 3M)—that MEIS1, HOXA11, c-Myc and BCL2 were significantly downregulated upon DNMT3A-knockdown (Fig. 3N-Q). Interestingly, GSEA analysis of RNA sequencing data in scr- but not DNMT3A-siRNA targeted OCI-AML2 tumors demonstrated significant enrichment for hallmark gene sets of oxidative phosphorylation (Fig. 3R) and c-Myc targets (Fig. 3T). To provide a better overview of these gene sets and to underscore possible interactions between genes, we performed STRING analysis followed by topological analysis using the CytoHubba plugin in the analysis platform Cytoscape (Fig. 3S and U), which results in a graphic visualization of the 15 highest ranking genes in both biological networks (oxidative phosphorylation: Fig. 3S; c-Myc targets: Fig. 3U; Additional file 1: Figure S4). Our data thus underscore that mutated DNMT3A-dependent regulation of genes is responsible for oxidative phosphorylation and c-Myc transcriptional programs as potential targets of DNMT3A and their contribution in leukemogenesis in these cells.

### αCD33-mAB-P/P-FLT3-siRNA treatment leads to significantly reduced tumor growth in a FLT3-ITD positive MV4-11 xenograft mouse model

**Fig. 4:**
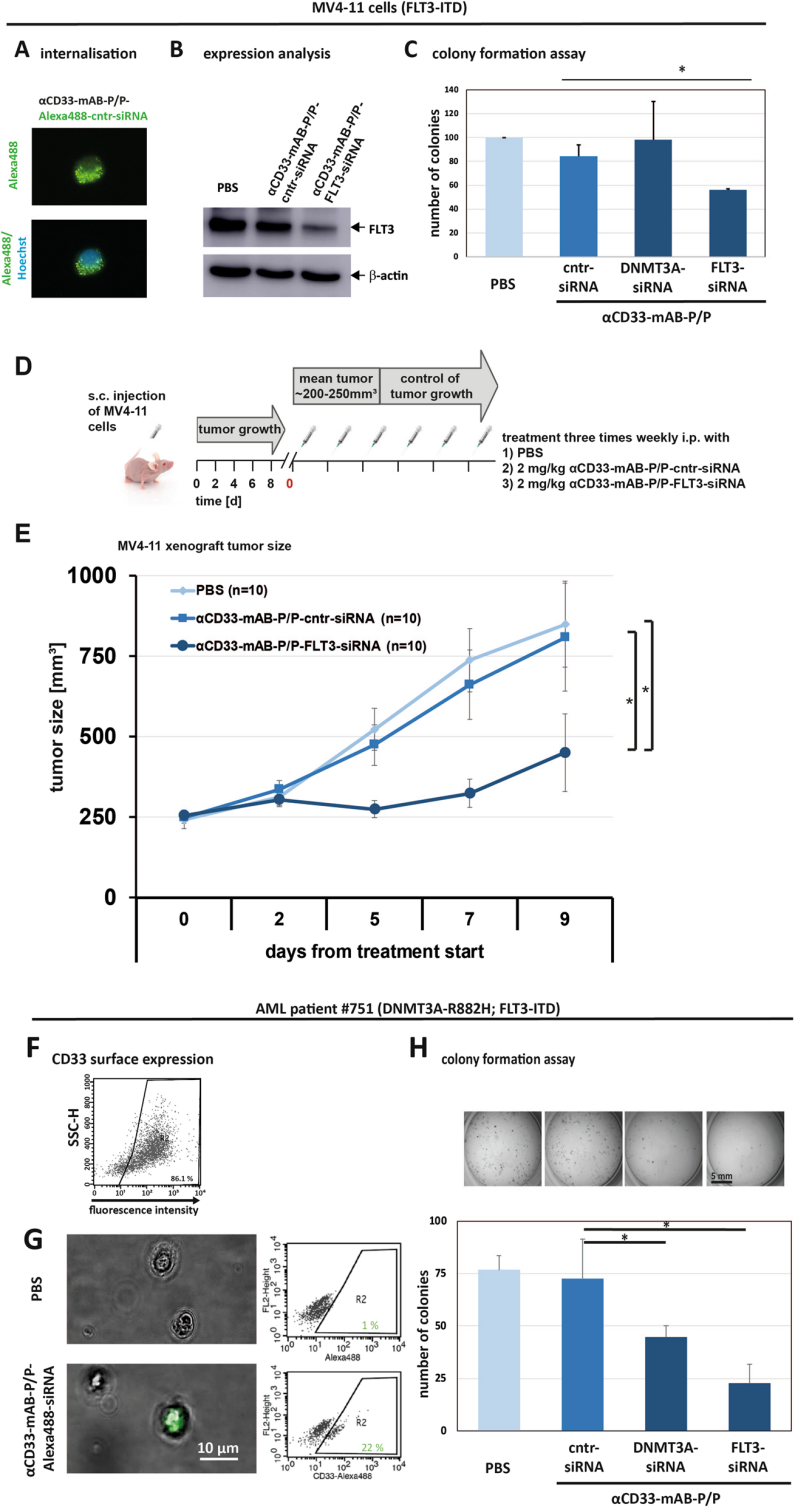
Knockdown of FLT3 via αCD33-mAB-P/P-nanocarrier leads to significantly decreased colony and tumor growth of MV4-11 cells and of colony formation of primary AML blasts. **A** Internalization of αCD33-mAB-P/P-Alexa488-control-siRNA into MV4-11 cells. **B** Western blot for FLT3 of FLT3-ITD-mutant AML cell line MV4-11. Expression of FLT3 was suppressed upon αCD33-mAB-P/P-FLT3-siRNA treatment in contrast to control-siRNA in MV4-11 with β-actin as control. **C** Colony formation assay of FLT3-ITD mutated cell lines MV4-11 (n = 3). There is a significant decrease in colony growth due to αCD33-mAB-P/P-FLT3-siRNA treatment in contrast to control (cntr)- or DNMT3A-siRNA carriers, respectively. Means plus SD of three independent experiments. 2-sided T-test: *p < 0.02. **D** Schematic overview of in vivo i.p. treatment after s.c. injection of 1 × 10^7^ MV4-11 cells in CD1-nude mice. Mice were treated with PBS, αCD33-mAB-P/P-control (cntr)-siRNA or αCD33-mAB-P/P-FLT3-ITD-siRNA three times weekly. **E** Tumor growth curves of MV4-11 transplants. Growth of MV4-11 tumors was significantly inhibited due to αCD33-mAB-P/P-FLT3-siRNA treatment in contrast to control-siRNA carrier treatment or PBS treatment. **F** Flow cytometric analysis of CD33-expression on the surface of AML patient #751 (driven by DNMT3A-R882H and FLT3-ITD mutations) cells. **G** The αCD33-mAB-P/P nanocarrier is able to internalize Alexa488-siRNA into patient blasts. Left: Fluorescence microscopy; right: Quantification via flow cytometry of non-treated (upper panels) and αCD33-mAB-P/P-Alexa488-control-siRNA internalized AML patient cells (lower panels). **H** Colony formation capacity of primary AML blasts. Cells were pre-incubated with the antibody-siRNA complex, resuspended in methylcellulose and cultivated for 8—12 days. Colonies were stained with INT, counted and photographed. There is a significant decrease in colony growth upon αCD33-mAB-P/DNMT3A-siRNA and αCD33-mAB-P/P-FLT3-siRNA treatment in contrast to control-siRNA carrier. Significance: *p < 0.05, 2-tailed T-test. Means plus SD of 3 independent replicates. α, anti

One of the most frequent mutations in AML is FLT3-ITD [23]. Moreover, DNMT3A-mutations and FLT3-ITD most likely cooperate as oncogenes in AML development [17, 39]. Therefore, a targeted therapy using siRNA may also include this important oncogene. We treated FLT3-ITD-bearing MV4-11 cells with our αCD33-mAB-P/P in complex with a mixture of two siRNAs and found that the complex was effectively internalized (Fig. 4A) into the MV4-11 cells and downregulated FLT3 (Fig. 4B). Colony formation was significantly decreased upon αCD33-mAB-P/P-FLT3-siRNA treatment of MV4-11 cells compared to αCD33-mAB-P/P-control-siRNA treated cells (Fig. 4C). This was not the case when DNMT3A-siRNA was delivered (Fig. 4C). We performed systemic in vivo treatment with αCD33-mAB-P/P-siRNA delivery in the FLT3-ITD-dependent MV4-11 xenograft in a CD1 nude mouse model (see Fig. 4D for schematic overview). The subcutaneous tumor growth was significantly reduced (*p* < 0.05) upon i.p. αCD33-mAB-P/P-FLT3-siRNA application, but not upon control siRNA and PBS application, which again underlines the effective transport and activity of the nanocarrier (Fig. 4E).

### Colony growth of primary AML blasts is reduced by treatment with αCD33-mAB-P/P and functional siRNAs

An in vitro standard to test a novel therapeutic strategy preclinically is the analysis of anchorage-independent colony growth of treated vs. non-treated primary patient cells. We chose an AML patient sample (patient #751), which harbored both, a FLT3-ITD and a DNMT3A-R882H driver mutation (Fig. 4). Around 86% blasts of patient #751 expressed CD33 on their surface as detected by flow cytometric analysis (Fig. 4F). To investigate internalization efficiency of αCD33-mAB-P/P-siRNA carriers, primary blasts were incubated o/n at 37 °C with αCD33-mAB-P/P complexed to Alexa488-labeled control-siRNA, and internalization was verified by fluorescence microscopy (Fig. 4G, lower panel).

Next, blasts were treated with αCD33-mAB-P/P with “payloads” of DNMT3A-siRNA or FLT3-siRNA, respectively. In contrast to αCD33-mAB-P/P-cntr (control)-siRNA, a significant reduction of colony formation could be detected after treatment with αCD33-mAB-P/P-DNMT3A-siRNA or αCD33-mAB-P/P-FLT3-siRNA (Fig. 4H). We substantiated the results of the in vitro-treatment of primary patient cells with three additional patient leukemia cells, patient #805 (DNTM3A- and FLT3-ITD-mutant), patient #770 (only DNMT3A-mutant) and #719 (FLT3-ITD) (Additional file 1: Figure S3). These results imply that 1) primary leukemic cells internalize the antibody–protamine-siRNA carriers, 2) the siRNA-mediated knockdown was efficient and 3) FLT3-ITD and DNMT3A-mutated blasts depend on both oncogenic factors to survive.

### Safety

To evaluate the effect of the RNAi in normal hematopoietic cells, CD34^+^/CD19^−^ peripheral blood mononuclear cells (PBMCs) of healthy persons were analyzed in colony formation assays (Additional file 1: Figure S5 G). A slight inhibition of colony growth caused by treatment with αCD33-mAB-P/P-siRNA for both, the cntr-siRNA and the functional siRNA carriers, could be detected (Additional file 1: Figure S5 G) probably representing an antibody-only effect. On the other hand, the differences between control-siRNA and FLT3-siRNA or DNMT3A-siRNA carrier treatment were not significant, indicating that normal PBMC are not specifically sensitive to FLT3 or DNMT3A knockdown in contrast to AML blasts (Additional file 1: Figure S5 G). Treatment with equimolar antibody concentrations of gemtuzumab-ozogamicin (GO, Mylotarg^R^), however, led to a significant reduction in colony growth of normal PBMC (Additional file 1: Figure S5 G). This indicated that treatment with αCD33-mAB-P/P-siRNA is less toxic for normal human hematopoietic cells than gemtuzumab-ozogamicin.

In addition, blood urea nitrogen (BUN) and serum creatinine (CR) values of mice treated with αCD33-mAB-P/P-siRNA carriers were determined from blood samples drawn from mice treated as described (see Additional file 1: Figure S5). The treatment with αCD33-mAB-P/P-siRNA carriers had no effect on BUN (Additional file 1: Figure S5 A) or CR (Additional file 1: Figure S5 B) values compared to values of mice treated with PBS. Moreover, mouse weight was unchanged under treatment (Additional file 1: Figure S5 E–F). In an independent OCI-AML2 xenograft transplant and treatment experiment, also liver enzymes AST (aspartate aminotransferase; other name: glutamic oxaloacetic transaminase (GOT)) and ALT (alanine aminotransferase; other name: glutamic pyruvic transaminase (GPT)) in mouse serum were unchanged when compared to values from saline control treated animals (Additional file 1: Figure S5 C-D), BUN and CR were reproduced as unchanged (not shown). Together, this shows that anti-CD33-mAB-P/P-siRNA carriers are not causing major clinical or laboratory toxicity in mice.

As some siRNAs can activate TLR as adverse effects, we further substantiated safety with TLR activation tests by in vitro assays that allow examination of NF-κB pathway activation via TLR2-signaling and of IRF pathway activation via TLR3 signaling (Additional file 1: Figure S9) in the monocytic reporter cell line THP1-DUAL designed for this purpose. While recommended positive controls such as poly(I-C) and Pam3CSK4 robustly induced TLR2 and TLR3 signaling, respectively, no nanocarrier combination treatment except αCD33-mAB-P/P-NRAS-siRNA induced a significantly elevated signal for induction. The latter is probably due to a cellular stress reaction, because THP-1 cells are NRAS mutated and dependent from this oncogenic signaling pathway. Therefore, a general TLR activation effect of αCD33-mAB-P/P-siRNA nanocarriers was not observed.

### Physical characterization of the nanocarriers

**Fig. 5:**
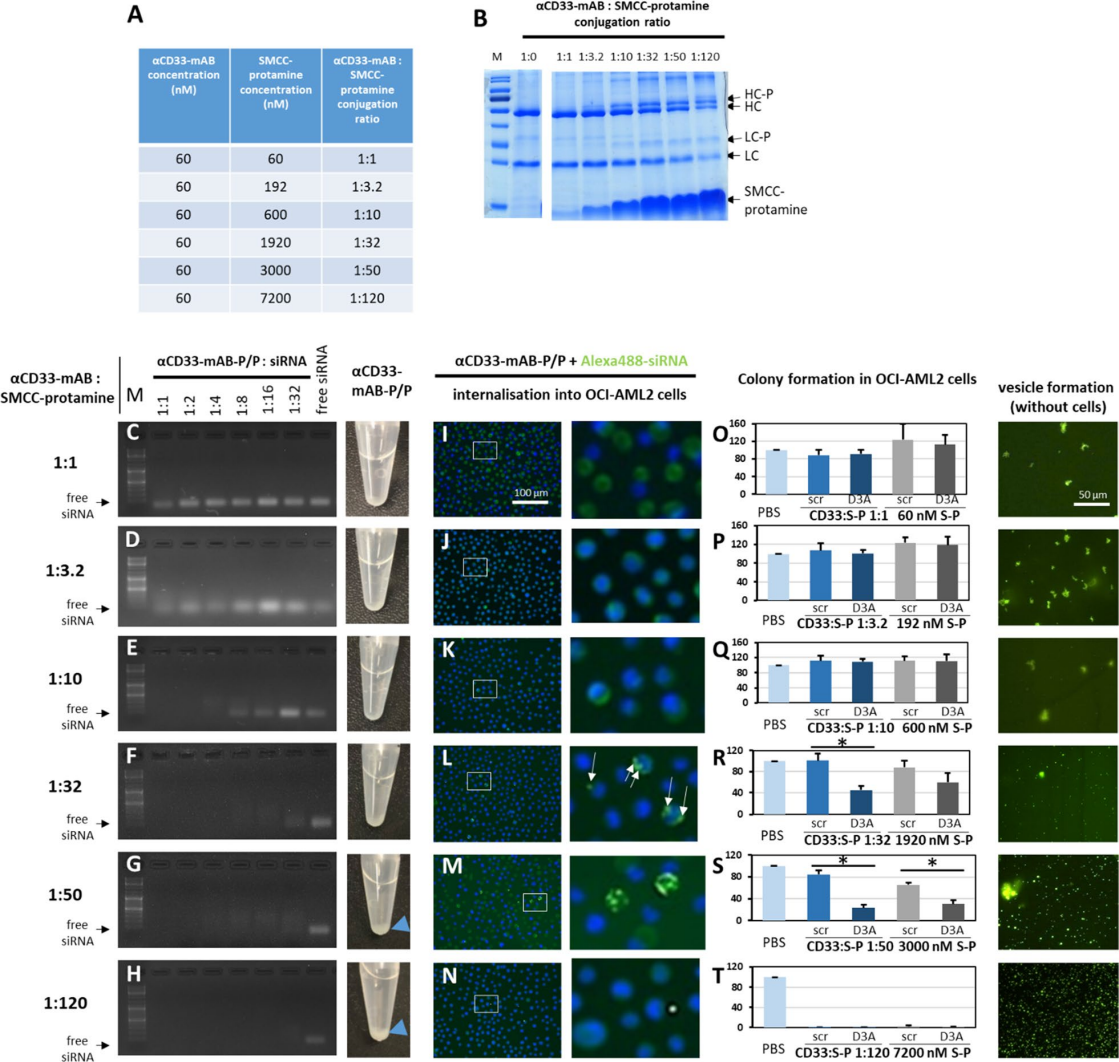
Attributes of effective αCD33-mAB:protamine conjugation ratios. **A** Concentrations tested and resulting molar ratios of αCD33 antibody (αCD33-mAB) to SMCC-protamine for the effective conjugation of both components. **B** Coomassie-stained SDS–PAGE showing uncoupled αCD33-mAB compared to the conjugation products that were coupled as depicted in A. The formation of a protamine-conjugated heavy chain (HC-P) and light chain (LC-P) showed an optimum at a 1:32 conjugation ratio with no further increase at higher ratios. **C–H **Left: Band-shift assays exhibiting siRNA binding capacity. Right: αCD33-mAB-P/P stored at 4 °C for several days shows precipitation at the bottom of the tube only at ratio 1:50 and 1:120 (blue triangles). **I-N** Internalization of Alexa488-control-siRNA complexed αCD33-mAB-P/P into OCI-AML2 cells. Complexes of αCD33-mAB-P/P transport Alexa488-siRNA into cells (left panel rectangles), with detailed magnifications (right panels). At conjugation ratios of 1:1–1:10 (I-K) only diffuse greenish background can be detected. **O-T **Left: Colony formation assays of the different conjugations in OCI-AML2 cells. Significance: *, *p* < 0.05, 2-tailed T-test. Means plus SD of three independent experiments. D3A, DNMT3A siRNA. S-P, SMCC-protamine**. **Right: αCD33-mAB-P/P-Alexa488-siRNA complexes form vesicles with different size and efficacy in presence of rising amounts of free SMCC-protamine. No vesicle formation at ratios of 1:1–10 (right: O-Q), apart from some unspecific aggregates (right side in P). α, anti

**Fig. 6:**
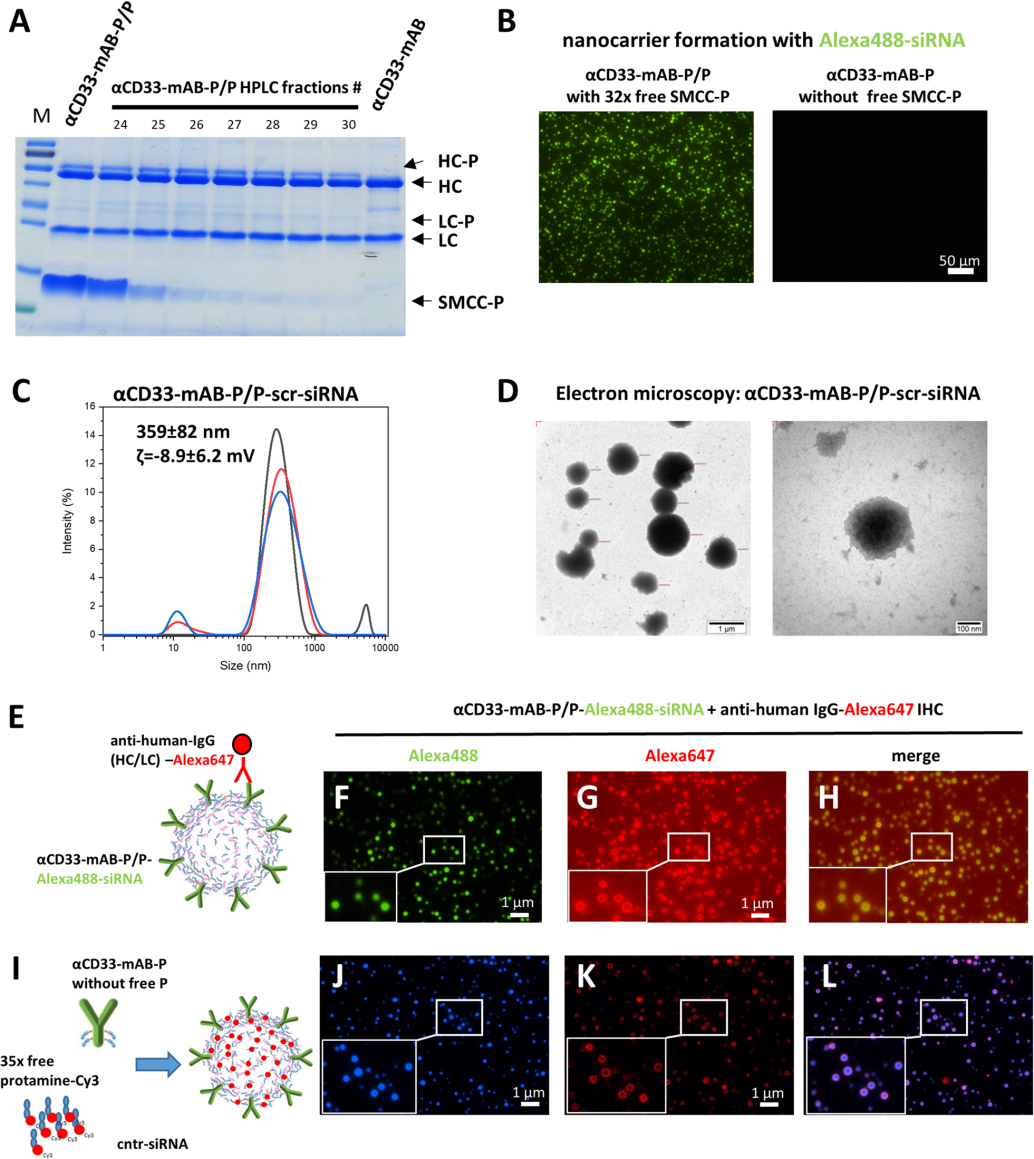
The αCD33-mAB-P/P nanocarriers only transport siRNA in presence of free SMCC-protamine (SMCC-P). **A** Coomassie-stained SDS–PAGE showing αCD33-mAB, αCD33-mAB coupled with SMCC-P and HPLC-fractions 29–30 of αCD33-mAB-P/P upon effective depletion of unbound SMCC-P; HC = heavy chain, LC = light chain, -P = SMCC-protamine. **B** Antibody–protamine conjugates with fluorescent Alexa488-siRNA in cell-free incubation overnight on chamber slides. αCD33-mAB-P/P with free SMCC-P forms visible vesicular structures (left panel), while αCD33-mAB-P after depletion of free SMCC-P (fraction 30, see A) do not form visible structures (right panel). **C.** DLS and zeta-potential measurement of αCD33-mAB-P/free protamine-scr-siRNA carriers. **D.** αCD33-mAB-P/P-scr-siRNA nanoparticles were left to form for 2 h and subjected to electron microscopy on copper grids by phosphotungstate negative staining. **E–H.** Immunostaining of nanocarriers with an anti-human IgG antibody to illustrate the accessibility and location of the αCD33-mAB in the outside rim and the siRNA in the lumen of the αCD33-mAB-P/P-nanocarriers. **I.** Protamine was chemically coupled to Cy3 and then incubated with αCD33-mAB-P that was depleted from free protamine and with non-fluorescent control-siRNA to form nanocarriers. This complexation was performed for 2 h at RT and nanocarriers were then immobilized o/n on slides for immunostaining as in G. **J.** αCD33-mAB-P/P-Cy3-control-siRNA show homogeneous Cy3 (blue) micelles. **K.** The same vesicles as in L show anti-human IgG-Alexa647 (red) fluorescence in ring-like structure around each vesicle (staining as depicted in E). **L.** Overlay of panels J and K. α, anti

Having obtained these therapeutic and safety results, we further analyzed the properties of nanocarrier formation and function for different conjugation ratios (Fig. 5) and in absence of free (SMCC-)protamine (Fig. 6 and Additional file 1: Figure S7). To this end, we conjugated molar ratios from 1:1 to a 1:100 (Fig. 5A) excess of SMCC-protamine over αCD33-mAB, which showed different coupling efficiencies as checked by the gel-electrophoretic properties of the resulting conjugates (Fig. 5B). Efficient coupling of heavy (HC) and light (LC) chain of the αCD33-mAB only appeared at ratios of  > / = 1:10 (Fig. 5B), which was in line with siRNA binding (Fig. 5C–H, left side). Interestingly, at ratios of 1:50 or higher, precipitation was visible (Fig. 5G, H, right side, compared to C-F, right side). Internalization of fluorescence-tagged siRNA into CD33-expressing OCI-AML2 cells (Fig. 5I–N) was seen with conjugates with a molar excess of 32 or 50 mol SMCC-protamine over αCD33-mAB.

As a functional analysis of the different conjugation ratios, we treated OCI-AML2 cells with different αCD33-mAB-P/P with control (scrambled, scr) versus anti-DNMT3A (D3A)-siRNA, respectively, and cultured equal numbers of cells in methylcellulose for anchorage-independent colony growth indicative for tumorigenicity. Of note, only the 1:32 (Fig. 5R) and 1:50 conjugates (Fig. 5S) showed a specific functional impact of DNMT3A-siRNA versus control siRNA with 50% less colony formation, compared to non-functional control siRNA. In contrast, compositions with lower excess such as 1:1 up to 1:10 showed no impact of DNMT3A-knockdown on colony growth (Fig. 5O–Q). Furthermore, concentrations of free SMCC-protamine yielding ratios higher than 1:50 led to cellular toxicity unrelated to the nature of the applied siRNA (Fig. 5T).

Further, when the αCD33-mAB-SMCC-protamine (αCD33-mAB-P) was conjugated with free SMCC-protamine at ratios of 1:1 up to 1:10, no efficient cell-free vesicle formation could be observed (Fig. 5O–Q, right side). At the ratios of 1:32 to 1:100, the vesicle formation was abundant (Fig. 5R–T, right side). Taken together, results shown in Fig. 5 suggest an optimal nanoparticle formation of αCD33-mAB-P to free SMCC-protamine ratio of 1:32 or 1:50, corresponding to the efficient siRNA delivery and internalization into cells without or only with little (1:50) precipitation or non-specific toxicities.

To confirm 1) that free (SMCC-)protamine is necessary for the proper formation of the nanocarrier and 2) that it is not the free (SMCC-)protamine, which mediates the cargo internalization but the antibody-receptor binding, as we saw in our recently published [6] and unpublished studies, we removed all excess free SMCC-protamine (“SMCC-P”) from the reaction mixture by preparative size exclusion chromatography or by protein G interaction chromatography (Fig. 6 and Additional file 1: Figure S7). Protamine-conjugated αCD33-mAB-P without free SMCC-protamine (Fig. 6A, fraction 29/30) was neither able to bind siRNA in a band-shift assay (Additional file 1: Figure S7 A), nor to internalize into CD33-positive OCI-AML2 cells (Additional file 1: Figure S7 D-E compared to B-C). Also, free SMCC-protamine alone could not transport Alexa488-siRNA into OCI-AML2 cells (Additional file 1: Figure S7 F-G). Vesicular nanocarrier formation was not detectable with αCD33-mAB-P without free SMCC-protamine (Fig. 6B). Moreover, neither colony formation of OCI-AML2 cells treated with αCD33-mAB-P after depletion of free SMCC-P and added to DNMT3A-siRNA was inhibited (Additional file 1: Figure S7 I), nor when treated with free SMCC-protamine with DNMT3A-siRNA (Additional file 1: Figure S7 J) when compared to added scr-siRNA or PBS, respectively. This confirmed the necessity of an intact αCD33-mAB-P/P nanoparticle in complex with siRNA to form what we now call αCD33-mAB-P/P-nanocarrier (Additional file 1: Figure S7 H).

To elucidate whether siRNA is needed to form vesicles, we incubated αCD33-mAB-P/P with rising amounts of Alexa488-control-siRNA (Additional file 1: Figure S7 K). Here, nanocarriers are efficiently formed with a molar excess of siRNA of 5–10 times over the antibody (Additional file 1: Figure S7 K).

Once formed properly by auto-assembly, the nanocarriers demonstrated a high stability toward pH shifts between pH 4.8 and 8.0 (Additional file 1: Figure S7 L) and different serum concentrations (Additional file 1: Figure S7 M). The αCD33-mAB-P/P-nanocarrier possesses a distinct size range of 359 ± 82 nm as determined by dynamic light scattering spectroscopy DLS (Fig. 6C), which was confirmed by electron microscopy (Fig. 6D). Here, spheroid, electron dense structures were determined by phosphotungstate negative staining (Fig. 6D).

We subsequently characterized the nanocarrier by immunostaining the human IgG proportion of the carrier complex (schematic overview in Fig. 6E) and observed a strong staining of the nanostructure rim (Fig. 6G), which revealed a position of the targeting antibody facing outward, while the Alexa488-tagged siRNA fills the lumen of the structure (Fig. 6F and H).

To further characterize the nanocarrier structure we also investigated the actual position of the protamine as the main electrostatic connector. Theoretically, free protamine could also form 1) a shell that is filled with siRNA or 2) siRNA and protamine could be distributed equally, as it was hypothesized before [40]. We therefore conjugated free protamine with Cy3 as a traceable chromophore (Additional file 1: Figure S8) and combined it with the αCD33-mAB-P depleted from free SMCC-protamine (as described in Fig. 6A and B) as well as with non-fluorescent siRNA to establish nanocarriers (Fig. 6I). We identified a luminal fluorescence staining of protamine-Cy3 (blue fluorescence in Fig. 6J), which closely resembled the pattern of the siRNA-Alexa488 (Fig. 6F and H compared to Fig. 6J and L). Moreover, immunostaining again with an anti-human IgG-Alexa647 antibody showed a ring-like staining surrounding the protamine-Cy3 containing vesicle (Fig. 6K and L). We conclude that the auto-assembly process forms a spheroid structure with the targeting IgG facing outward and a balanced composition of siRNA and free protamine filling the lumen of the nanocarrier sphere (Fig. 6H and L).

In summary, these characterization experiments show a sufficient corridor for nanocarrier production according to Good Manufacturing Practice (GMP) guidelines necessary for translation into early clinical trials.

### Treatment of patient-derived xenotransplanted (PDX) AML with αCD33-mAB-P/P-siRNA nanocarriers

**Fig. 7:**
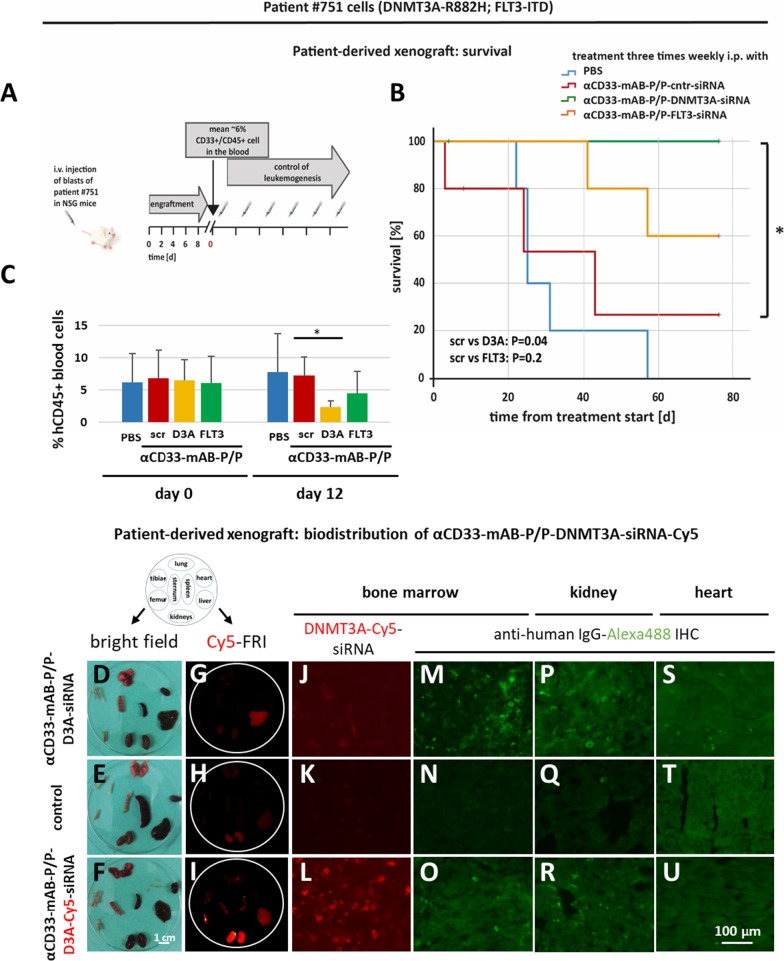
Knockdown of DNMT3A in a PDX-AML model leads to significantly increased survival. **A** Schematic overview about in vivo treatments after i.v. injection of 1 × 10^7^ blasts of DNMT3A-mut/FLT3-ITD-mut patient #751 AML into NSG mice. Mice were treated by i.p. injection with PBS, 4 mg/kg αCD33-mAB-P/P-control (scrambled: “scr”)-siRNA, αCD33-mAB-P/P-DNMT3A-siRNA or 4 mg/kg αCD33-mAB-P/P-FLT3-siRNA three times weekly (n = 5 in each group). **B** Kaplan–Meier survival curves: Treatment with αCD33-mAB-P/P-DNMT3A-siRNA led to survival benefit for all mice within this setting, αCD33-mAB-P/P-FLT3-siRNA showed a trend. **C** αCD33-mAB-P/P-DNMT3A treatment significantly reduces hCD45-positive circulating leukmia cells in mouse blood. Day 12 was chosen, because control mice were still alive. FLT3-siRNA treated mice showed the same trend. **D-U** In the same PDX model, engrafted animals were treated with αCD33-mAB-P/P-DNMT3A nanocarriers with Cy5-labeled siRNA (lower row) or non-labeled (upper row) as well as PBS control (middle row). **D, E, F.** Bright field photographs of Cy5-FRI in **G, H, I** Mice treated with αCD33-mAB-P/P-nanocarriers loaded with Cy5-labeled siRNA showed Cy5 signals in human leukemia engrafted bone marrow (**I**, **L**) as well as excretion by kidney (**I**), while no or very weak signals were detected in non-labeled siRNA treated (**G**) and PBS controls (**H**). **M-U** Organ sections immunostained for human IgG showed distinct signals in αCD33-mAB-P/P -nanocarrier-treated, but not control mice in bone marrow (αCD33-mAB signal) and kidney (excretion), but not in irrelevant organs such as heart. α, anti; D3A, DNMT3A; FRI, Fluorescence reflectance imaging

The analysis of primary cells directly obtained from patients and xenotransplanted into immune-deficient mice represents a preclinical tumor model close to the patient’s situation and is a standard in the development of therapy options. To this end, we established a patient-derived AML xenograft (PDX) transplantation of blood AML mononuclear cells in NSG mice using the same patient sample as analyzed in vitro before (Fig. 4F–H). We transplanted cells from AML patient #751 (see Fig. 4F–H) first as a primary transplantation to expand the sample, then followed by secondary intravenous (i.v.) transplantation into NSG mice. We checked for engraftment of the leukemic blasts by flow cytometry of blood samples (Fig. 7A). When AML was established, indicated by a mean of 6% CD33-positive cells in the blood of transplanted mice, treatment was started. Moribund mice were euthanized and data analyzed as survival curve (Fig. 7B). Treatment with αCD33-mAB-P/P-DNMT3A-siRNA nanocarriers lead to significantly prolonged survival of PDX-transplanted mice compared to PBS- or αCD33-mAB-P/P-scr-siRNA treated mice (Fig. 7B). Treatment with nanocarriers transporting FLT3-siRNA showed a clear trend toward longer survival, which with the numbers of animals used did not reach significance (Fig. 7B). On the other hand, the treatment with the DNMT3A and FLT3-siRNA nanocarriers reduced the percentage of circulating hCD45-positive leukemia cells, which was compared on day 12 after treatment (Fig. 7C). This was without signs of toxicity as determined by mouse weight (Additional file 1: Figure S5 H). These experiments underline the efficacy of the αCD33-mAB-P/P nanocarrier and the importance of mutated DNMT3A for the survival of the leukemic cells used. Moreover, flow cytometry of remaining CD33-positive human blasts in the blood and bone marrow over time revealed loss of this target marker on the cell surface in αCD33-mAB-P/P-treated mice for prolonged time (details not shown). This illustrates that our αCD33-mAB-P/P-nanocarrier internalizes very efficiently also in vivo into all target cells in the hematopoietic system, which we further analyzed in more detail.

### Biodistribution of nanocarrier treatments

To perform preliminary studies on the biodistribution of our αCD33-mAB-P/P-siRNA nanocarriers, we ordered Cy5-labeled DNMT3A-siRNA and formed red-fluorescent nanocarriers. First, we treated mice carrying subcutaneous OCI-AML2 tumors at the end of the treatment with PBS as depicted in Fig. 2F. We injected them i.p. with nanocarriers complexed with Cy5-labeled DNMT3A-siRNA, prepared the tumor and different organs 24 h after injection (Additional file 1: Figure S6 A and B) and subjected them to ex vivo-fluorescence imaging. Cy5-labeled siRNA nanocarrier-treated mice revealed significant enrichment of Cy5-related fluorescence signals in the large OCI-AML2 tumors (Additional file 1: Figure S6 D compared to C), as well as in excretion organs such as kidney (in some animals) and – to a lesser extent—liver, but not in irrelevant organs such as heart, lung, bone marrow and spleen (Additional file 1: Figure S6 D). Moreover, upon cryosectioning, only αCD33-mAB-P/P-DNMT3A-siRNA-Cy5 treated tumors show a distinct red-fluorescent signal (Additional file 1: Figure S6 G), non-fluorescently labeled siRNA treated (αCD33-mAB-P/P-scr-siRNA, Additional file 1: Figure S6 F) as well as PBS treated mice (Additional file 1: Figure S6 E) did not show signals in the respective tumors. Next, we immunostained cyrosections with αhIgG-Alexa488-antibody that detects the human αCD33-carrier antibody and found respective signals in nanocarrier-treated (Additional file 1: Figure S6 I, J), but not in untreated tumors (Additional file 1: Figure S6 H) as well as in kidney glomeruli (Additional file 1: Figure S6 L and M), but not in irrelevant tissues such as heart (Additional file 1: Figure S6 N-P and data not shown).

In a second set of experiments, PDX-transplanted and engrafted mice were treated with αCD33-mAB-P/P-nanocarriers complexed with DNMT3A siRNA, which was conjugated with Cy5 chromophor as described above. After termination of the experiment, mice were sacrificed and organs prepared and subjected to *ex vivo* Cy5 fluorescence analysis (Fig. 7D-I). In mice treated with Cy5-labeled siRNA, bone marrow of femurs as well as kidneys revealed enriched Cy5 fluorescence (Fig. 7I, compared to G and H) in ex vivo as well as on cryosectioned slides (Fig. 7L, compared to J and K). When bone marrow, kidney and heart cryosections were immunostained for presence of human IgG (the humanized anti-CD33-carrier antibody gemtuzumab), we detected αhIgG-Alexa488 signals in bone marrow (Fig. 7M and O) and kidneys (Fig. 7P and R), but only background staining in heart (Fig. 7S and U). In non-antibody control treated mice, bone marrow and kidney revealed no human IgG-related signals (Fig. 7N, Q and T, respectively). We conclude that after i.p. injection, nanocarriers containing the carrier IgG as well as the siRNA are transported in vivo through the blood stream to xenografted cells of human origin expressing the CD33 antigen, may this be a subcutaneous tumor or an i.v.-engrafted primary leukemia. Part of the nanocarriers seems to be excreted by kidney, which will be subject of further detailed investigations in preparation of a clinical study.

### αCD33-mAB-P/P nanocarriers loaded with ibrutinib-Cy3.5 inhibit clonal leukemic cell growth

The full potential of our nanocarrier technology platform to target, e.g., CD33-positive AML cells can further be demonstrated by exchanging the siRNA component by an anionic small molecule. As an example, we recently generated an anionic form of the uncharged approved BTK-inhibitor ibrutinib that we electrostatically loaded into a αCD20-mAB-P/P nanocarrier and showed efficient targeting of CD20-positive DLBCL lymphoma [6]. This supports that our nanocarrier can not only be adapted to the desired target cell by choice of the internalizing antibody and by the definition of an siRNA against a respective oncogene, but also by the complexation of other molecules rendered anionic.

**Fig. 8:**
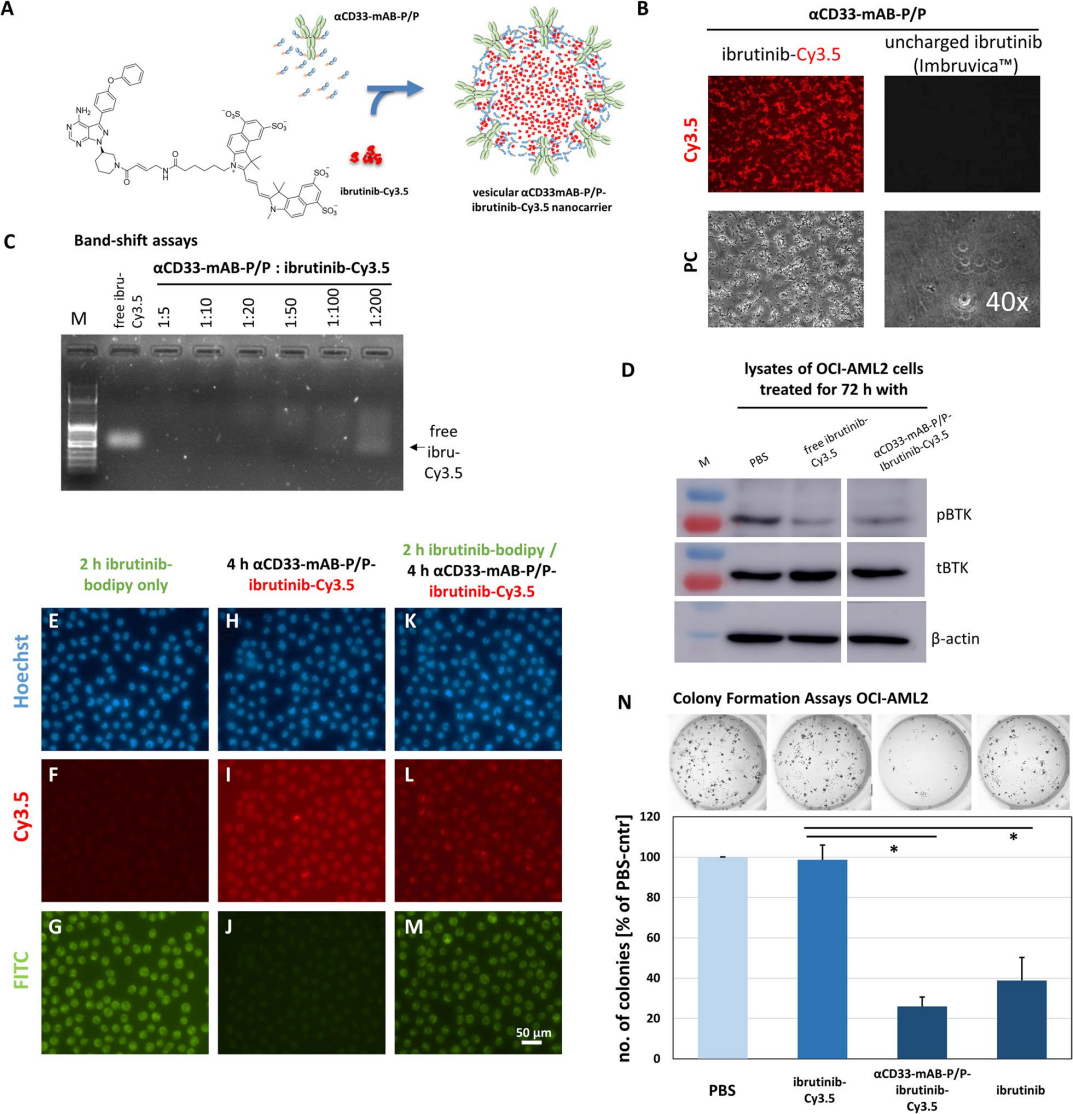
Cellular targeting of Bruton’s kinase (BTK) by αCD33-mAB-P/P-ibrutinib-Cy3.5 and inhibition of clonal growth of treated AML-cells. **A** Schematic overview: spontaneous assembly of the αCD33-mAB-P/P-ibrutinib-Cy3.5 nanocarrier. **B** The αCD33-mAB-P/P conjugate was incubated for 2 h with anionic ibrutinib-Cy3.5 (left panels) or uncharged ibrutinib (trademark: imbruvica™; right panels) in 1:20 ratio and applied to cell-culture treated glass slides for fluorescence microscopy. Only αCD33-mAB-P/P-ibrutinib-Cy3.5 complexes led to the formation of numerous vesicles, where the larger vesicles showed intense Cy3.5 fluorescence (upper left panel) and vesicle formation in phase contrast (PC, lower left panel). No nanocarrier formation in presence of uncharged ibrutinib (upper and lower right panels, bubbles in lower right panel are mounting air inclusion artifacts). **C** Electromobility shift assays showing the electrostatic loading capacity of ibrutinib-Cy3.5 to conjugates from A in a molar ratio. One mol of αCD33-mAB-P/P can bind at least 20–50 mol ibrutinib-Cy3.5. **D** CD33-positive OCI-AML2 cells were treated by the respective conjugates shown for 72 h, lysed and subjected to SDS–PAGE and Western blotting for phospho-BTK (pBTK), total BTK (tBTK) and actin as a loading control. Both, free ibrutinib-Cy3.5 and αCD33-mAB-P/P-ibrutinib-Cy3.5 complexes inhibited the phosphorylation of BTK. **E-M** Fluorescence microscopy of OCI-AML2 cells treated with targeting conjugates and controls showing a marked intracellular enrichment of Cy3.5-signals (I). Fluorescence microscopy of OCI-AML2 cells pre-treated with ibrutinib-bodipy (green, G and M) do not show intracellular enrichment of Cy3.5-signals after αCD33-mAB-P/P-ibrutinib-Cy3.5 treatment (J compared to G). **N** Upper panels: Photographs of representative colony formation assays as summarized in the lower panel. In colony formation assays, 1200 nM untargeted ibrutinib-Cy3.5 did not reduce colony growth of OCI-AML2 cells, while the specifically targeted αCD33-mAB-P/P-ibrutinib-Cy3.5 (60 nM nanocarrier: 1200 nM ibrutinib-Cy3.5) reduced the colony growth to below 30% of the PBS controls, more than the treatment with 1200 nM uncharged ibrutinib (right-most bar). Significance: *, *p* < 0.05, 2-tailed T-test. Means plus SD of 3 independent experiments. α, anti

The application of ibrutinib in AML, predominantly in combination with cytotoxic agents such as decitabine or azacytidine, as well as BCL2 inhibitors such as venetoclax is currently under consideration [24, 25]. Here, we tested the effectivity of the strongly anionic derivative of ibrutinib, ibrutinib-Cy 3.5 to form an αCD33-mAB-P/P nanoparticle that can be safely applied to target the CD33 expressing AML cell line OCI-AML2. The ibrutinib-Cy3.5 chemical synthesis is published in [6]. Figure 8A depicts the structure. Ibrutinib-Cy3.5 with αCD33-mAB-P/P spontaneously forms a nanoparticle exposing red fluorescence in cell-free conditions, in contrast to unmodified ibrutinib, which shows no nanocarrier formation (Fig. 8B). Due to its strong anionic charge (- 4) and small molecular weight, up to 100 mol ibrutinib-Cy3.5 per mol of αCD33-mAB-P/P carrier construct can be complexed, as seen by electro-mobility shift assays (Fig. 8C), revealing an enormous cargo to carrier ratio. When treated with carrier-complexed and non-complexed ibrutinib-Cy3.5, OCI-AML2 cells showed reduced autophosphorylation of the ibrutinib main covalent binding target protein, the Bruton’s kinase (BTK) under both conditions (Fig. 8D). This indicated a target hit. We then performed a “competition” experiment, in which CD33-positive OCI-AML2 cells were treated first with a competitively binding, green fluorescent ibrutinib-bodipy [6] derivative and secondly with αCD33-mAB-P/P nanocarrier loaded with the red-fluorescent ibrutinib-Cy3.5. Here, OCI-AML2 cells treated with the αCD33-mAB-P/P-ibrutinib-Cy3.5 nanocarrier showed a reduction of cellular Cy3.5-dependent staining (Fig. 8L) compared to those cells only treated with free ibrutinib-Cy3.5 (Fig. 8I), which indicates target competition between both ibrutinib derivatives and confirms a convergent mode of action by covalent BTK-binding.

To evaluate the functional consequences of αCD33-mAB-P/P-ibrutinib-Cy3.5 nanocarrier treatment in AML cells, we performed colony formation assays. OCI-AML2 cells treated with αCD33-mAB-P/P-ibrutinib-Cy3.5 showed a massive reduction of colony numbers to approx. 20% of those treated with the free ibrutinib-Cy3.5, indicating a much higher anti-tumor activity of the αCD33-mAB-P/P-nanocarrier-mediated enrichment of the compound, but comparable to the commercially available ibrutinib (Fig. 8N).

## Discussion

### RNAi and cancer

Cancer can be understood as a disease based on genomic alterations and the formation of malignant networks formed by the interaction of those [41]. Therefore,definition of vulnerable nodes, which are indispensable to tumors, led to a plethora of potential target genes [42], among them those that can not be addressed by classical therapies [43]. Owing the unique feature of regulating gene expression, RNAi has been proposed to be an ideal strategy for cancer treatment [44] since RNAi may be able to revert the malignancy-driving networks mentioned. Given its precision as compared to classic small molecular drugs, RNAi is widely used in tumor gene therapy research and led to the discovery of oncogenic networks by genome wide screens [45][45].

Tumor-indispensable candidate genes have been discovered by performing experiments in one or more of the following areas: 1) directly inhibiting oncogenes, 2) interference with tumor suppressor genes or tumor anti-apoptosis genes, 3) interference with the tumor´s signal transduction pathways, 4) inhibition of tumor angiogenesis-related factors and 5) reduction of tumor drug resistance. RNAi not only helped unravel those five “achilles heels” of tumors, it consequently has the potential to act as a therapeutic principle itself. One of the advantages of RNAi technology is the rapid development and its intrinsic flexibility by the simple exchange from one sequence of interest to another or even the combination of them. Because gene expression and cell proliferation are closely linked in cancer, oncology is one of the primary objectives of RNAi-based treatment [46],

On the other hand, discoveries elucidating the molecular and cellular biology of the T cell, especially its capacity for antigen-directed cytotoxicity, has become a central focus for engaging the immune system in the fight against cancer [47]. This led to new strategies, including immune checkpoint blockade, CAR-T-cell technology and cancer vaccines. However, with growing experience in this rapidly evolving field, limitations become visible, for instance acquired resistance by evasion [48], accumulation of mutations in genes regulating T-cell recognition [49] and up-regulation of anti-apoptotic factors [50]. Taken together, although immunotherapies translated successfully into clinical applications, RNAi therapy has the potential for another significant contribution to cancer therapy.

### RNAi drug development: state of the art

Although RNAi drug development was highly praised in the scientific community, clinical results with RNAi drugs often fell short of expectations mainly because of the inability to provide two key necessities for in vivo application: a) protection against degradation and immune detection and b) selective transport into the intended cell. The concepts presented since either provide solely a protection of siRNA in form of nanoparticles, lipid nanoparticles (LNPs), polymers, dendrimers, nucleic acid nanostructures or exosomes [7, 8] polymeric matrixes [51], or enhanced the cellular targeting by the use of common targeting ligands for siRNA including aptamers, antibodies, peptides [52] and small molecules such as GalNAc [53]. However, the first set of applications lacked targeting and the latter adaptations, while enhancing cellular targeting, exposed incomplete protection of siRNA in comparison to complexation or encapsulation methods. Nevertheless, approval of siRNA drugs such as patisiran (Onpattro^R^) composed as a nanocapsule decorated with the protein ApoE that mediates uptake into the liver, where transported siRNA can knockdown transthyretin to treat patients with hereditary transthyretin-mediated amyloidosis [54, 55] shows the general power of RNAi.

### A new electrostatic nanocarrier platform.

With the antibody–protamine/P/siRNA nanocarriers presented here, we offer a solution concerning the protection of siRNA by electrostatic complexation. Moreover, we establish a modular system of decoration with targeting antibodies, which provides specific internalization of the nanocarriers into the intended target cell and ensures the transport of a much higher siRNA cargo-to-carrier ratio than direct antibody-(si)RNA conjugates (ARCs). Since the underlying universal electrostatic conjugation mechanisms are so versatile, they are not restricted to the complexation of nucleic acids, but can also be applied to complex other anionic molecules. The formulation of these nanocarriers is technically simple, optimum ratios of components assemble spontaneously in aqueous solution and do not require advanced protein chemistry, complex microfluidics for lipid nanoparticle encapsulation or polymer chemistry to provide shelter for the siRNA. In our system, we relied on protamine as the universal electrostatic connector: protamine is a highly cationic peptide displacing histones from the DNA during spermatogenesis [56] capable of condensing DNA to almost crystalline densities [57]. Phylogenetically, protamines evolved from primitive histone ancestors [58] and are evolutionary highly adapted for a strong, but reversible nucleic acid binding. Hence, protamine has been proposed for the complexation of siRNAs before [18, 59]. However, those reports either relied on a linear carrier molecule antibody–protamine:siRNA exposing weak siRNA complexation of 1:1 or totally omit a targeting moiety leading to a mere transfection effect of certain protamines. The antibody–protamine/free protamine/siRNA nanocarriers presented here are formed in an auto-assembly process simply by providing specific corridors of relative concentrations in order to ensure a proper electrostatic balance. Within this auto-assembly process, the comparably bulky targeting IgGs are forced facing outward of the nanocarrier sphere shell, while chemically conjugated protamines keep the siRNA/free protamine core structure protected. This leads to a stable spheroid structure that is capable of recognizing its binding partners in form of extracellular domains of specific cell surface molecules allowing cellular internalization. At the same time, this novel therapeutic principle forms a safe harbor around the siRNA bulk cargo.

Without having the detailed information about the complex structure of the nanocarriers presented here, we have used this system [3–5] using the epidermal growth factor receptor (EGFR) antibody cetuximab to target EGFR-positive colorectal cancer cells and inhibit their growth by RNAi against their major targets KRAS and PIK3CA. These targets are still regarded as “undruggable” except the rare KRAS-G12C variants [60].

### Adaptation of the siRNA nanocarrier system to AML

Here, we applied our modular siRNA therapeutic delivery system a) to CD33-positive AML cells and b) to target oncogenic DNMT3A and FLT3 by siRNA. By coupling siRNA to the anti-CD33 antibody gemtuzumab via protamine, we established a successful vesicular nanocarrier system to target AML cells. DNMT3A gene expression was downregulated in vitro using DNMT3A-siRNA, resulting in decreased viability of the DNMT3A-mutant AML cell lines and patient cells. In addition, AML cell colony formation could be specifically reduced. In vivo, a significantly reduced tumor growth could be detected, as well as reduced proliferation and increased apoptosis in the tumor cells.

### Mutated DNMT3A as an oncogenic driver of AML

For a plethora of reasons, DNMT3A might be a key component of leukemogenesis and therefore, an important primary target for therapeutic interventions: DNMT3A mutations occur in ~ 25% of AML patients and are associated with a poor prognosis [11, 12, 62]. Although the effects of mutations in DNMT3A are largely unclear to date, it has been shown that malignant hematopoietic stem cell expansion is induced by mutated DNMT3A and, together with an NPM1 mutation or FLT3-ITD, it promotes anthracycline resistance [11]. On the other hand, there are hints in literature of seemingly paradox functions of DNMT3A [63] as a potential tumor suppressor, for instance in lung tumors [64], the physiological relevance of this is still a matter of debate. In hematological malignancies, mutant DNMT3A function is better understood: The most common mutation is the DNMT3A-R882H mutation [12]. It has a dominant negative effect on DNA methylation and reduces it by up to 80% [11, 14]. It was shown, that DNMT3A-R882-mut induces cytokine-independent growth of TF-1 cells and the maintenance of this phenotype relies on continuous presence of DNMT3A-R882-mut [65]. In addition, the R882H mutation leads to a significant change in gene expression, leading to a lack of differentiation and growth advantage of undifferentiated AML blasts. It was demonstrated that DNMT3A-R882-mut-dependent AML cells are specifically susceptible to DNMT inhibitors such as azacytidine [66]. Last, DNMT3A mutations are indicators for a shorter overall survival in AML patients over 60 years, and notably in those with intermediate-risk cytogenetics [61]. Here, we prove that inhibition of DNMT3A in DNMT3A-mutated cells leads to growth inhibition, while normal blood cells are not dependent on DNMT3A expression. The higher susceptibility to azacytidine methyl transferase inhibitor, the higher risk stratification and our findings classify mutated DNMT3A as a driver mutation whose inhibition by our RNAi approach seems a suitable therapeutic option. Interestingly, AML cell lines with wild type DNMT3A, but also other malignant transformations do not react to knockdown of DNMT3A.

### A path to ARCH development prevention?

Interestingly, DNMT3A mutations belong to the heavily debated age-related clonal hematopoiesis (ARCH) mutations [67, 68]. Identical mutations characterize overt AML in the elderly individual as driver events. The most frequent ARCH mutations are mutated IDH1, IDH2, TP53, DNMT3A, TET2 and ASXL1 genes, they are recurrent in healthy elderly individuals exhibiting clonal hematopoiesis. Although their association to AML development remains correlative, it is tempting to speculate that the early detection and direct inhibition of early mutations such as DNMT3A-R882H might lead to the leukemia prevention by knockdown or extinction of the mutated cell clones.

### Inactivating oncogenic FLT3

There are multiple clinical studies on the impact of FLT3-ITD inhibitors such as sorafenib, midostaurin, quizartinib and crenolanib on AML [69]. Since 2017, the tyrosine kinase inhibitor midostaurin has been approved for the treatment of AML carrying FLT3-ITD [70, 71]. However, many of these small molecules have to be applied in doses close to toxicity, since they are not targeted to AML cells. Moreover, lower dosage may trigger the evolution of mutated cancer cells to develop resistance to this binding, e.g., by mutation of the binding site and consequently the AML becomes refractory to the respective therapy. With the development of the antibody–protamine/P-cargo platform technology, we propose a therapeutic alternative in two ways: 1) the antibody-P/P-carrier can potentially amplify the effect of a small molecular inhibitor in the target cell as shown with ibrutinib-Cy 3.5 for lymphoma [6] and to a lesser degree here for AML. The nanocarrier effectively targets cells of interest, allowing higher anti-tumor effectivity at much lower doses and avoids toxicity by non-specific sequestration. 2) For oncogenes not druggable, a downregulation by RNAi becomes an option for an individualized therapy again without much sequestration and off-target effects.

The study presented here shows that anti-CD33-antibody–protamine nanocarriers complexing anionic anti-cancer cargo such as siRNA or small molecules could represent a future-oriented possibility for leukemia therapy. The double specificity of the technology could lead to less toxicity by effective targeting, and RNAi against DNMT3A as well as other mutations driving AML could be used for individual patients. Application of this carrier technology also for prevention and therapy of other human diseases seems possible.

## Supplementary Information

**Additional file 1.** Supplement figures (Figs. S1–S9), supplemental references.

## Data Availability

The RNAseq datasets generated and analyzed during the current study are available from the corresponding author on reasonable request.

## Abbreviations

A549: Human NSCLC cell line
ALT: Alanine aminotransferase
AML: Acute myeloid leukemia
AST: Aspartate aminotransferase
BTK: Bruton’s tyrosine kinase
BUN: Blood urea nitrogen
CD20: Cell surface molecule
CD33: Cell surface molecule
CHO-S: Chinese hamster ovary cells, suspension
CR: Serum creatinine
DLBCL: Diffuse-large B-Cell lymphoma
DLS: Dynamic light scattering
DNMT3A: DNA methyl transferase 3A
EGFR: Epidermal growth factor receptor
FLT3: Fms like tyrosine kinase 3
FRI: Fluorescence reflectance imaging
GFP: Green fluorescent protein
GO: Gemtuzumab-ozogamicin, Mylotarg®
GOT: Glutamic oxaloacetic transaminase
GPT: Glutamic pyruvic transaminase
GSEA: Gene set enrichment analysis
HC: Heavy chain
IRES: Internal tandem duplication
ITD: Internal tandem duplication
KG1: Human AML cell line
LC: Light chain
mAB: Monoclonal antibody
MV4-11: Human AML cell line
NSCLC: Non-small cell lung cancer
NSG: NOD.Cg-Prkdcscid Il2rgtm1Wjl/SzJ mouse strain
OCI-AML2: Human AML cell line
OCI-AML3: Human AML cell line
OCI-Ly19: Human DLBCL cell line
P: Protamine
PDX: Patient-derived xenograft transplantation
RNAi: RNA interference
Scr: Scrambled
siRNA: Small inhibiting RNA
