# Supplementary material for: Electrostatic anti-CD33-antibody–protamine nanocarriers as platform for a targeted treatment of acute myeloid leukemia
Source: J Hematol Oncol. 2022 Dec 1;15:171. doi: 10.1186/s13045-022-01390-5 (PMC9716776; doi:10.1186/s13045-022-01390-5)
Supplement: Supplementary file 1 — Additional file 1. Supplement figures (Figs. S1–S9), supplemental references. [file 13045_2022_1390_MOESM1_ESM.docx]

**Supporting Information**

**Electrostatic anti-CD33-antibody-protamine nanocarriers as platform for a targeted treatment of Acute Myeloid Leukemia**

*Nicole Bäumer, Annika Scheller, Lisa Wittmann, Andreas Faust, Mara Apel, Subbaiah Chary Nimmagadda, Christiane Geyer, Katharina Grunert, Neele Kellmann, Matthias Peipp, Sareetha Kailayangiri, Matias Ezequiel Gutierrez Suburu, Cristian A. Strassert, Mathias Schenk, Lilo Greune, Christian Rüter, Petra Dersch, Wolfgang Hartmann, Claudia Rossig, Dario Neri, Carsten Müller-Tidow, Christian Schwöppe, Christoph Schliemann, Cyrus Khandanpour, Georg Lenz, Wolfgang E. Berdel, Sebastian Bäumer*


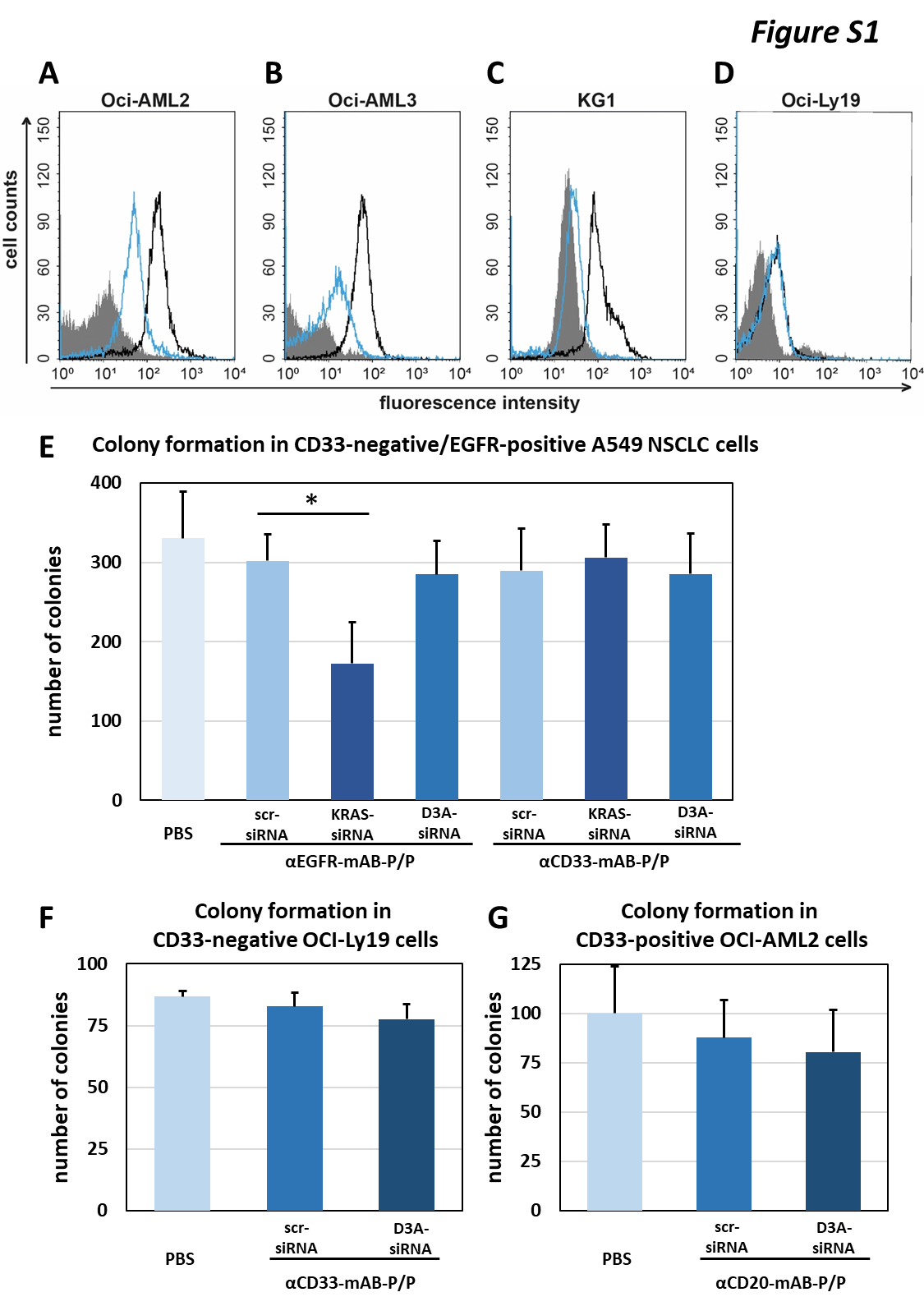


**Figure S1: Cell surface receptor and oncogene specificity of αCD33-mAB-P/P-siRNA-nanocarrier. A-D.** CD33 internalization provoked by αCD33-mAB-P/P binding. Flow-cytometric analysis of CD33 exposure on CD33-positive cell lines OCI-AML2 (A), OCI-AML3 (B) and KG1 (C) and as negative control on CD33-negative cell line OCI-Ly19 (D). Grey: unstained cells, black: αCD33-mAB stained cells to detect the CD33-expression on cell surface, blue: incubation with αCD33-mAB-P/P complex leads to less CD33 detection on CD33-positive cells due to internalization of the CD33 molecule with bound antibody (A-C), while not shift occurs in CD33-negative OCI-Ly19 cells. **E.** A549 NSCLC cells, which are dependent on oncogenic KRAS function, show a significant inhibition in colony formation, when functional KRAS-siRNA is incorporated into a relevant targeting αEGFR-mAB cetuximab-decorated protamine-nanocarrier, but the very same siRNA is ineffective when incorporated into an irrelevant non-targeting αCD33-mAB-P/P-KRAS-siRNA nanocarrier, because CD33 in not expressed in NSCLC cells. **F.** In CD33-negative OCI-Ly19, DNMT3A-siRNA showed no response in colony formation. **G.** In CD33-positive, CD20-negative OCI-AML2 cells dependent on DNMT3A action, the application of DNMT3A-siRNA incorporated into αCD20-mAB-P/P-nanocarrier exhibited no effect on colony formation. N=3 for all colony formation assays, P<0.05, T-test. α, anti; scr, scrambled; D3A, DNMT3A siRNA.


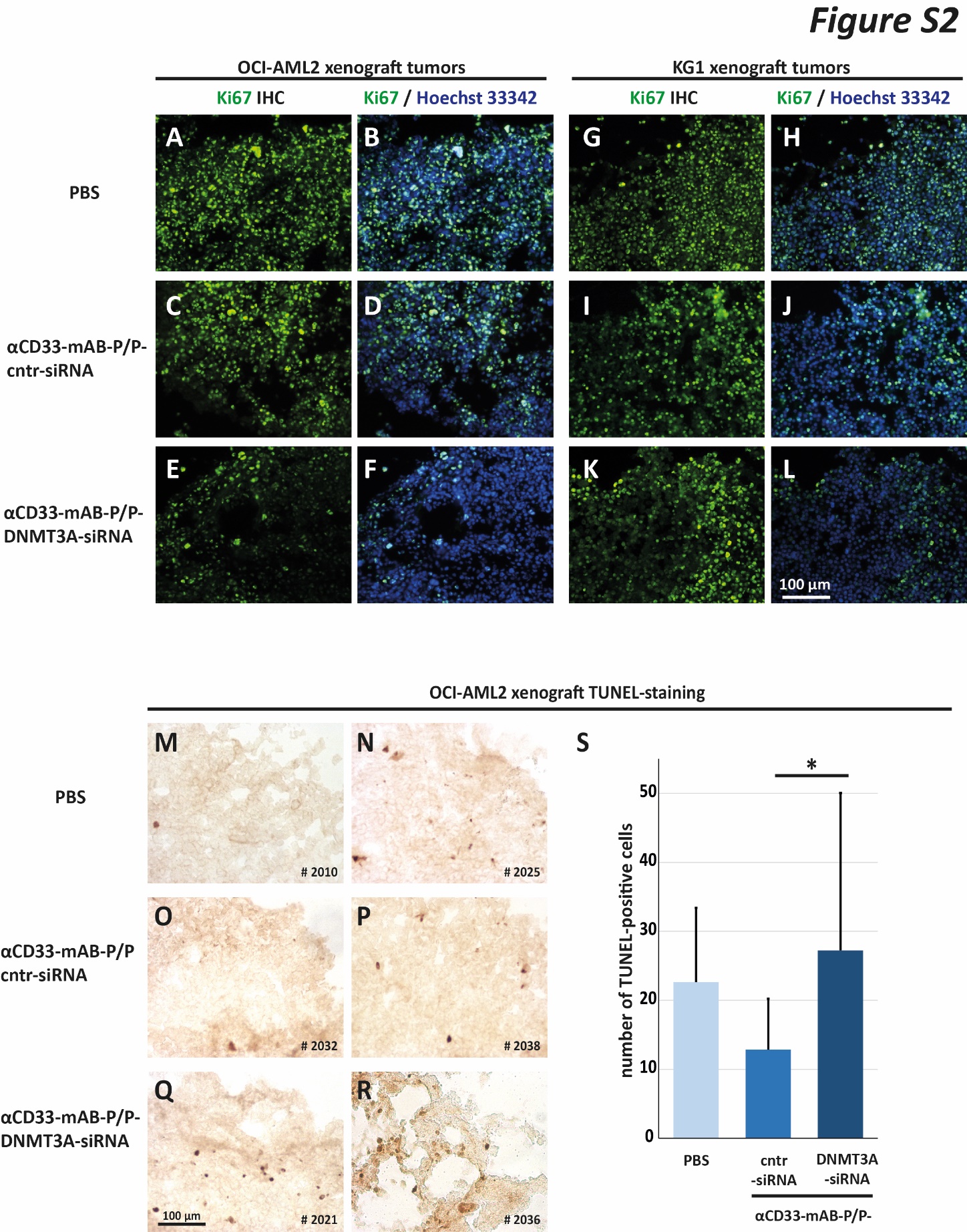


**Figure S2: *Ex vivo* examined OCI-AML2 xenografts show significantly lower proliferation rates and higher number of apoptotic cells after αCD33-mAB-P/P-DNMT3A-siRNA nanocarrier treatment as compared to PBS and control-siRNA nanocarrier treatment. A-G.** Immunofluorescence determination of proliferation marker Ki67 on histologic sections of OCI-AML2 (A-F) and KG1 (G-L) xenografts explanted at the end of experiments. In sections of OCI-AML2 tumors treated with αCD33-mAB-P/P-DNMT3A-siRNA nanocarriers the expression of proliferation marker Ki67 was markedly reduced (E and F). **M-R.** Immunohistologically detected apoptosis in OCI-AML2 tumor sections by TUNEL assay. The abundance of TUNEL positive apoptotic cells (dark brown) was markedly higher in OCI-AML2 tumor sections treated with αCD33-mAB-P/P-DNMT3A-siRNA nanocarrier (Q and R) as compared to PBS (M and HN or control-siRNA nanocarrier treatment (O and P). S. Statistic analysis of TUNEL positive nuclei in Oci-AML2 tumor sections. n=3; significance: *, P < 0,05. Numbers indicate individual mouse identifier. α, anti; cntr, control.


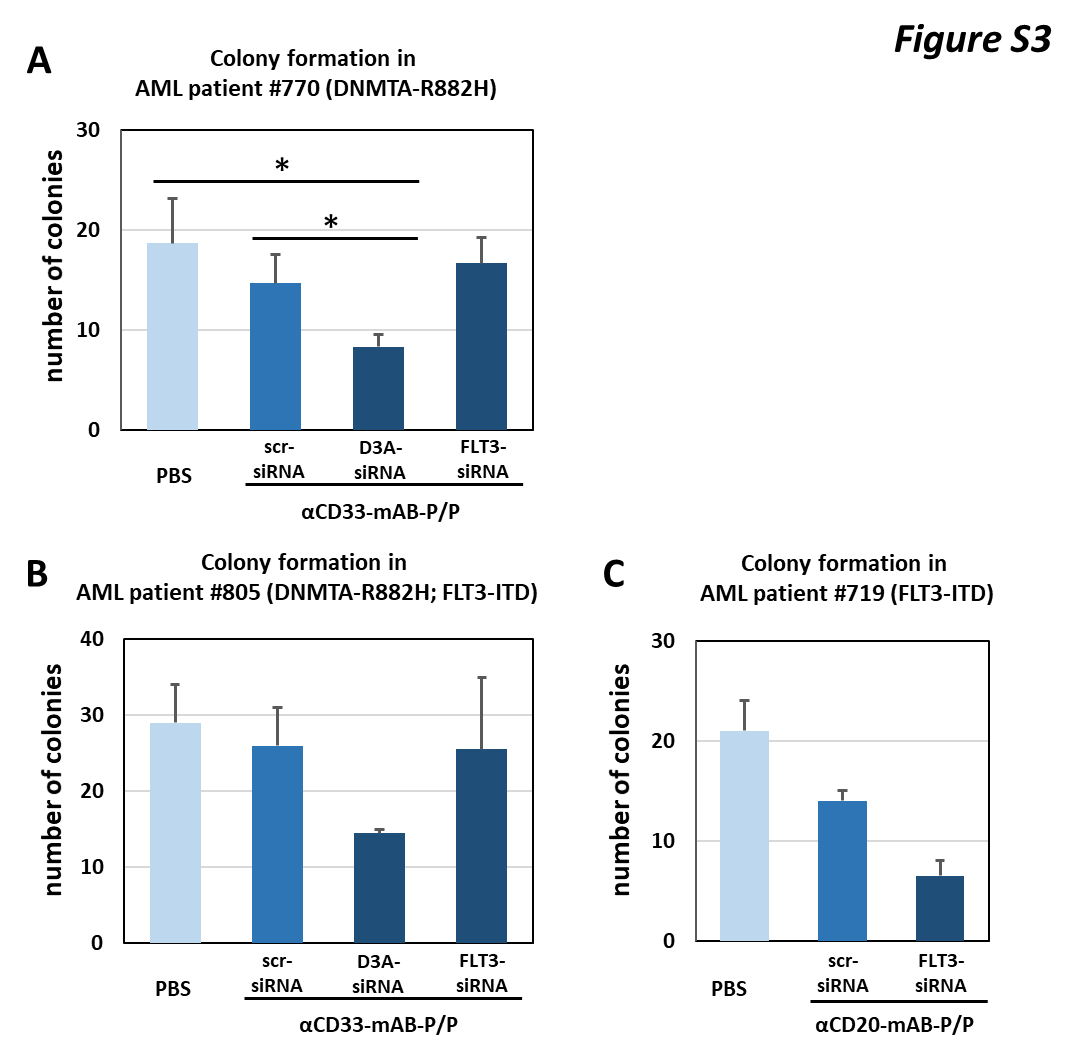


**Figure S3:** **DNMT3A-siRNA loaded αCD33-mAB-P/P-nanocarriers reduce colony formation in primary patient leukemia cells.** Patient cells were essentially cultivated and seeded and treated in colony assays as described in the Methods section. **A.** In DNMT3A-R882H-mutant patient #770, DNMT3A-specific siRNA, but not control (scr)- or FLT3-siRNA reduced colony growth by half. Shown here mean + SD of n=3, *, P<0.05, T-test. **B.** In DNMT3A-R882H-mutant patient #805, DNMT3A-specific siRNA, but not control (scr)- or FLT3-siRNA reduced colony growth by half. **C.** In the FLT3-ITD-mutant patient #719, application of FLT3-siRNA was shown to be effective. B-C: Due to limitations in primary cell material, here mean values + SD of duplicates are shown, therefore no further statistical analysis was performed. α, anti, D3A, DNMT3A; scr, scrambled.

**A**

| **Overlaps**  **(of 12)** | **Gene hub** | **Function** |
| --- | --- | --- |
| 11 | COX4I1  (Cytochrome c oxidase (COX) subunit 4I1) | A therapeutic target in the Treatment of Medullary Thyroid Cancer (1). |
| 10 | SDHB  (Succinate dehydrogenase complex, subunit B) | Loss of SDHB expression is frequently identified in hemangioblastoma of the central nervous system (2). Downregulated in clear cell renal cancer (3). |
| 9 | ATP5A1  (ATP synthase F1 subunit alpha) | TNK2 dependent phosphorylation of ATP synthase F1 subunit alpha has an important role in progression of prostrate cancers (4). |
| 9 | UQCRC2  (Ubiquinol-cytochrome-c reductase complex core protein 2) | Regulates epithelial-mesenchymal transition in Gastric Cancer (5). Promotes tumorigenesis by facilitating p53 degradation (6). |
| 9 | SDHA  (Succinate dehydrogenase complex, subunit A) | Loss of SDHA expression is identified in multiple myeloma patients (7). Myc-mediated SDHA acetylation regulated gene expression and tumorigenesis (8). |
| 9 | UQCRFS1  (Ubiquinol-cytochrome c reductase) | Overexpressed in ovarian carcinoma (9), gastric cancer and has a role in tumor development (10). |
| 9 | UQCRC1  (Ubiquinol-Cytochrome C Reductase Core Protein 1) | Elevated expression was observed in Pancreatic cancers and was correlated with poor prognosis of the disease (11). |
| 9 | CYC1  (Cytochrome C1) | Predicts poor response in breast cancer patients (12) and favourable prognostic marker in estrogen receptor-positive breast carcinoma (13). |
| 9 | ACO2  (Aconitase 2) | Inhibited proliferation of breast cancer cells by promoting mitochondrial oxidative metabolism (14). Loss of ACO2 promoted colorectal cancer progression (15). |
| 9 | ATP5H  (Mitochondrial membrane ATP synthase Complex V) | Loss of expression is associated with multimodal cancer therapy resistance (16). |

**B**

| **Overlaps**  **(of 12)** | **Gene hub** | **Function** |
| --- | --- | --- |
| 10 | CCT2  (Chaperonin Containing TCP1 Subunit 2) | CCT2 is an essential component of the chaperonin complex for tumorigenesis (17). Its expression is inversely correlated with survival in breast cancer patients (18). |
| 10 | RPLP0  (Ribosomal Protein Lateral Stalk Subunit P0) | Role in cell cycle arrest and cell apoptosis (19) and non-homologous end joining-mediated DSB repair(20) |
| 9 | CCT4  (Chaperonin Containing TCP1 Subunit 4) | A biomarker in head and neck squamous cancer. Overexpression of CCT4 is associated with lower overall survival (21). |
| 9 | NHP2  *(NHP2* Ribonucleoprotein) | A core component of the telomerase complex, overexpressed in tumors and predicts poor prognosis (22) and a role in DNA repair (23). |
| 9 | RPS3  (Ribosomal Protein S3) | A component of the 40S ribosomal subunit and affects colon cancer growth by modulating p53 and lactate dehydrogenease (24). Knockdown of RPS3 inhibited cell growth and induced apoptosis in breast cancer cells (25). |

**Figure S4:** Protein-Protein interaction network/subnetworks were analyzed using 12 algorithms of cytoHubba plugin for top 15 hub genes regulating (**A**) Hallmark_Oxidative phosphorylation and (**B**) Hallmark_Myc_Targets-V1 were presented. Hub genes representing intersection of at least nine algorithms, their function in various cancers were presented.


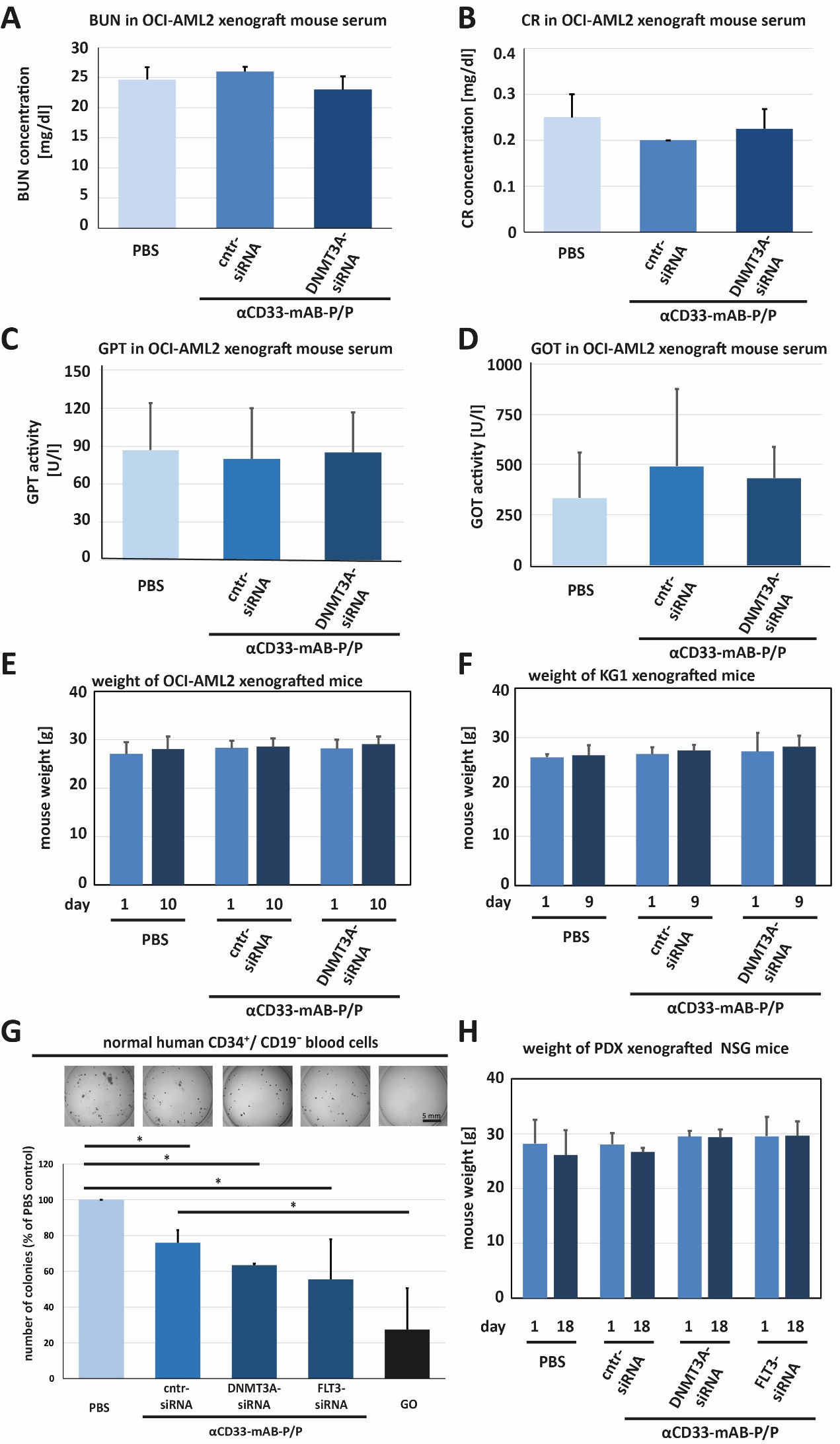


**Figure S5: αCD33-mAB-P/P-siRNA treatment has no *in vivo* toxicity on kidney function, on normal human primary blood cells, or visible in the body weight. A, B.** No significant changes could be observed in the blood urea nitrogen (BUN) or creatinine (CR) values in blood serum of mice treated with αCD33-mAB-P/P-DNMT3A-siRNA nanocarrier in comparison to PBS and αCD33-mAB-P/P-cntr-siRNA nanocarrier treated mice. **C, D.** In an independent experiment, no significant changes could be observed in the aspartate aminotransferase (AST; other name: glutamic oxaloacetic transaminase (GOT)) or alanine aminotransferase (ALT; other name: glutamic pyruvic transaminase (GPT)) values in serum of mice treated with αCD33-mAB-P/P-DNMT3A-siRNA (n=4) nanocarrier in comparison to PBS (n=5) or αCD33-mAB-P/P-cntr-siRNA (n=6) nanocarrier treated mice. **E-F.** In the *in vivo* experimental trial presented in Figure 2, mouse weight values were determined on each treatment day. Shown here are the values of day 1 and after the treatment on day 10 (C, OCI-AML2 trial) and day 9 (D, KG1 trial). There were no signs of toxicity in this global “clinical” parameter. **G.** The colony growth of CD34+/CD19- normal blood cells is reduced after treatment with αCD33-mAB-P/P-siRNA nanocarriers but without significant differences between cntr-siRNA, DNMT3A-siRNA or FLT3-siRNA carriers. This represents the known inhibitory effect of αCD33-mABs alone. In contrast, treatment with gemtuzumab-ozogamicin (GO) at equimolar antibody concentrations led to significantly reduced colony growth (n=3). Significance: *, P < 0.05. **H.** In the *in vivo* PDX experiment presented in Figure 7 mouse weight values were determined on each treatment day. Shown here are the values of day 1 and after the treatment on day 18. The values varied insignificantly in all groups showing no global “clinical” signs of toxicity. α, anti; cntr, control.


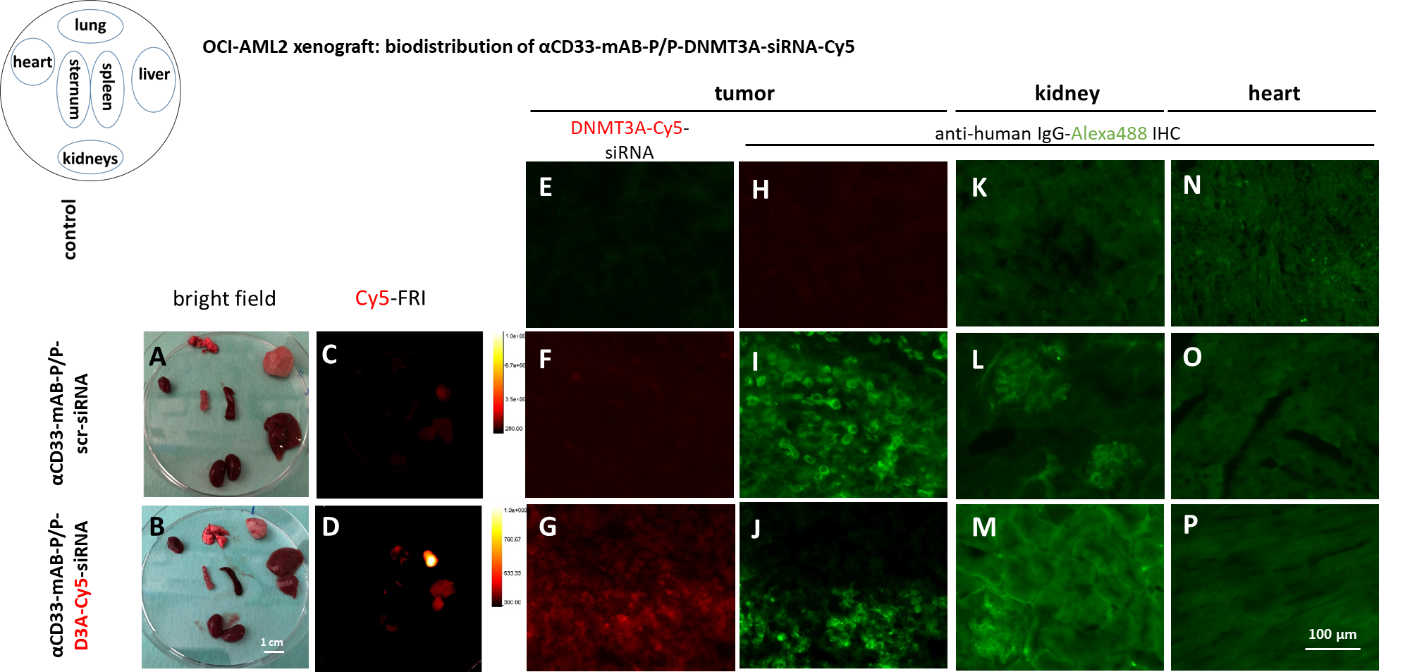


**Figure S6: Biodistribution of αCD33-mAB-P/P-siRNA nanocarriers in subcutaneous xenograft tumor model.** Mice carrying subcutaneous OCI-AML2 tumors were treated with nanocarriers complexed with Cy5-labelled DNMT3-siRNA as well as non-labelled control siRNA and PBS by subcutaneous injection and organs prepared 24 hrs after injection (**A** and **B**, schematic overview in the upper panel). Cy5-labelled siRNA treated mice *ex vivo* revealed significant enrichment of Cy5-related fluorescence signals in the large OCI-AML2 tumors (**D**), as well as in excretion organs such as liver and in some animals in the kidney (see also Figure 7 I), but not in irrelevant organs such as heart, while non-fluorescently labelled siRNA treated as well as PBS treated mice did not show signals in the respective organs (**C** and not shown). The Cy5 fluorescence was confirmed in cryosections (**G**, compared to **E** and **F**). Immunostaining of cyrosections with an αhIgG-Alexa488-antibody revealed respective signals in nanocarrier-treated (**I**, **J**), but not in control tumors (**H**) as well as in kidney glomeruli (**L**, **M**), but not in irrelevant tissues such as heart (**N-P**). α, anti; scr, scrambled control; FRI, Fluorescence reflectance imaging.

**
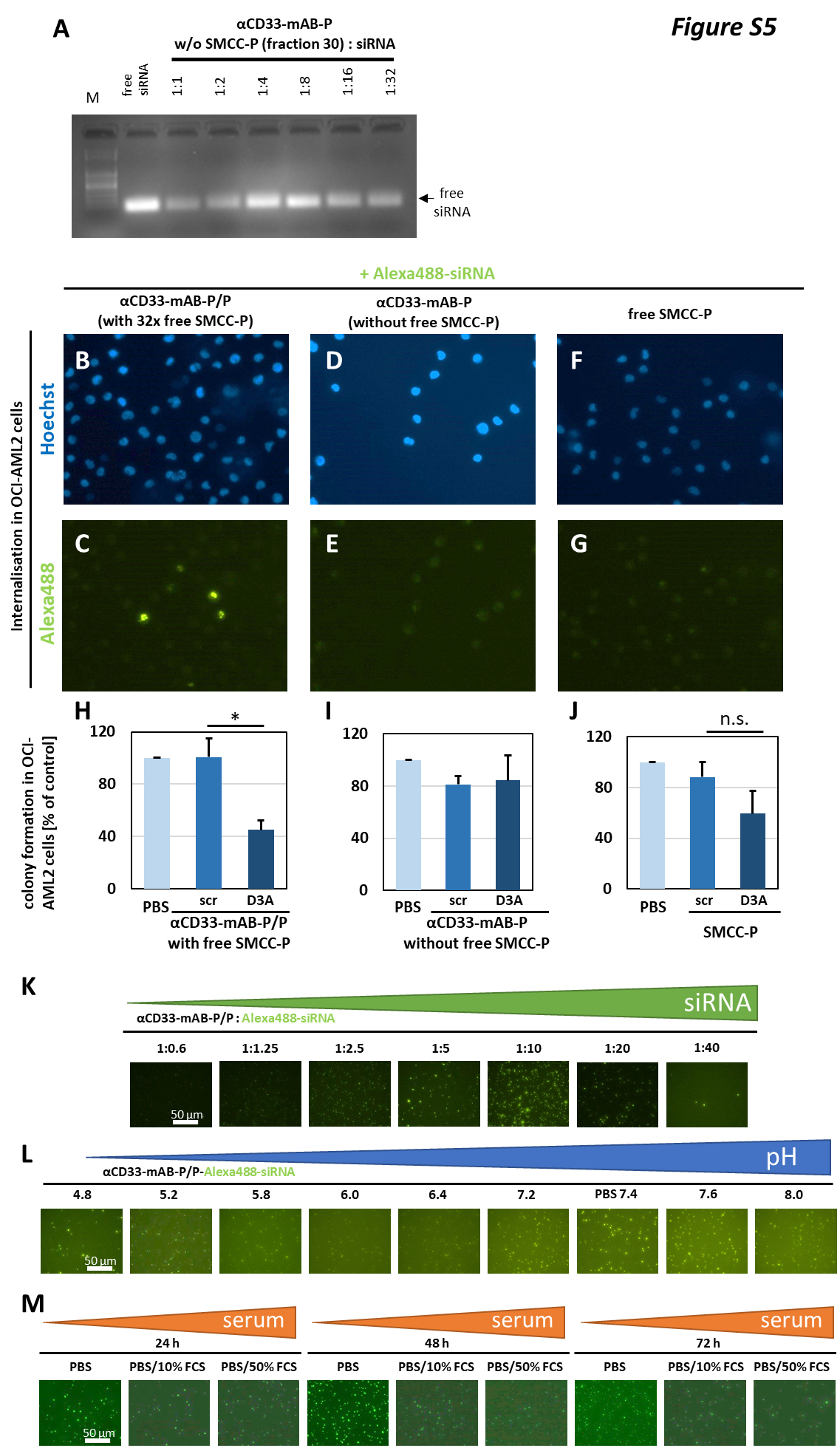
**

**Figure S7: The αCD33-mAB-P complexes only transport siRNA in presence of free SMCC-protamine (SMCC-P). A.** Band-shift assays of αCD33-mAB-P without free SMCC-P reveals no siRNA complexing in the absence of free SMCC-P. **B-G.** Internalization into OCI-AML2 cells of αCD33-mAB-P/P with free SMCC-protamine (**B-C**), lack of internalization without free SMCC-protamine (**D-E**) and SMCC-protamine only (**F-G**), all plus fluorescent Alexa488-control-siRNA. **H-J.** OCI-AML2 colony formation after incubation with αCD33-mAB-P/P with free SMCC-protamine (**H**), without free SMCC-protamine (**I**) and SMCC-protamine only (**J**), always either with control-siRNA (scr) or DNMT3A-specific siRNA (D3A) in comparison to PBS-treated cells. **K.** Rising amounts of fluorescent Alexa488-siRNA show an optimal nanocarrier formation at a molar ratio of αCD33-mAB-P/P to siRNA of 1:5 up to 1:10. **L.** αCD33-mAB-P/P**-**Alexa488-siRNA nanocarriers were stable upon incubation o/n in chamber slides containing buffers with different pH ranging from pH 4.8 to 8.0. **M.** Stability αCD33-mAB-P/P**-**Alexa488-siRNA nanocarriers after incubation for 24, 48 and 72 h in chamber slides in PBS/10% FCS and PBS/50% FCS compared to PBS. In the PBS photographs, the background to carrier contrast is more pronounced due to the absence of serum. α, anti.


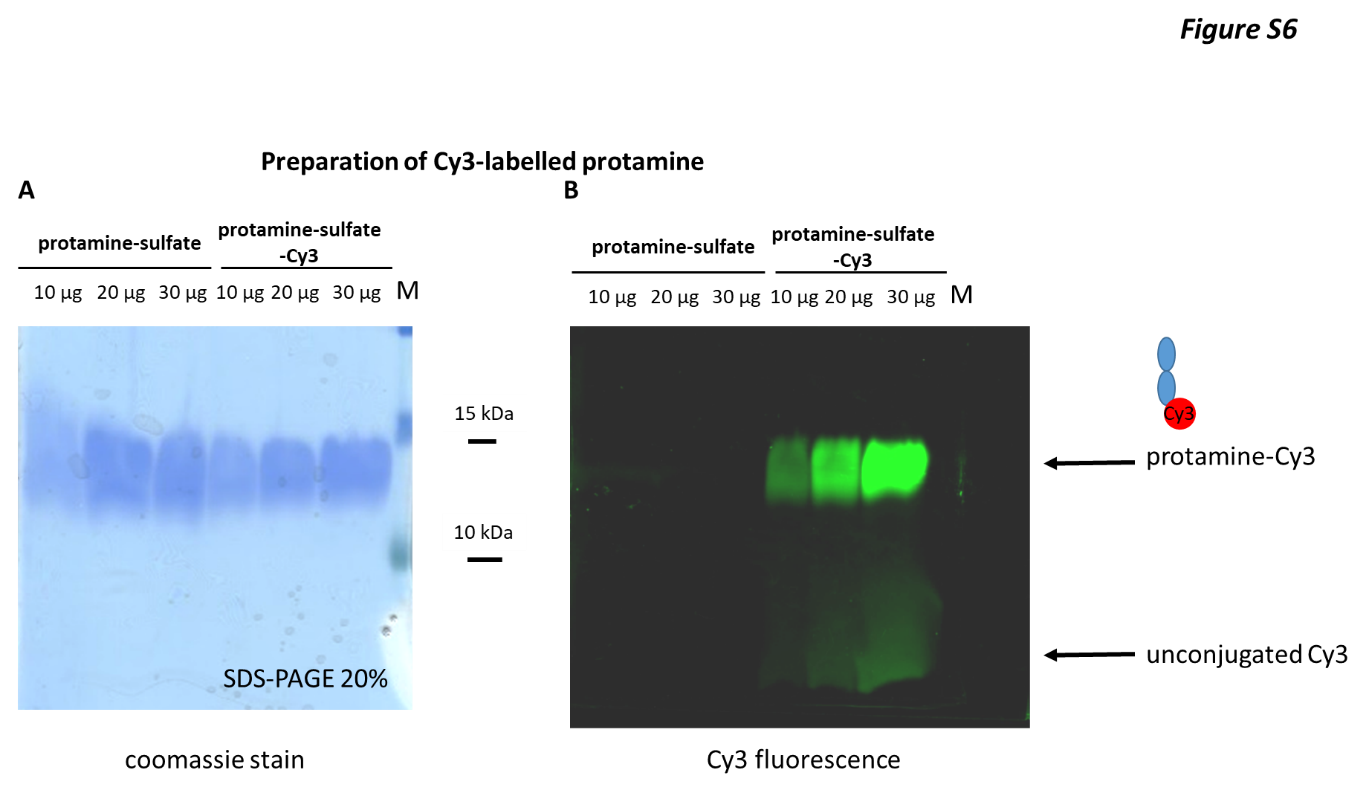


**Figure S8: Preparation of Cy3-labelled protamine. A.** Protamine-sulfate was solved in water and conjugated with activated Cy3 (Cytiva) according to the manufacturer’s recommendations, followed by a gel-filtration step in order to remove the majority of non-reacted activated Cy3. Non-labelled and Cy3-labelled protamine was subjected to 20% SDS-PAGE as indicated, followed by a Cy3-fluorescence scan on a gel imager (**B**). The fluorescently labelled protamine travelled at a band of approximately 12 kDa, the non-labelled protamine exhibited no fluorescence. After fluorescence inspection, the gel was stained with Coomassie (**A**). The fluorescently labelled protamine was used to form the nanocarriers presented in Figure 8.


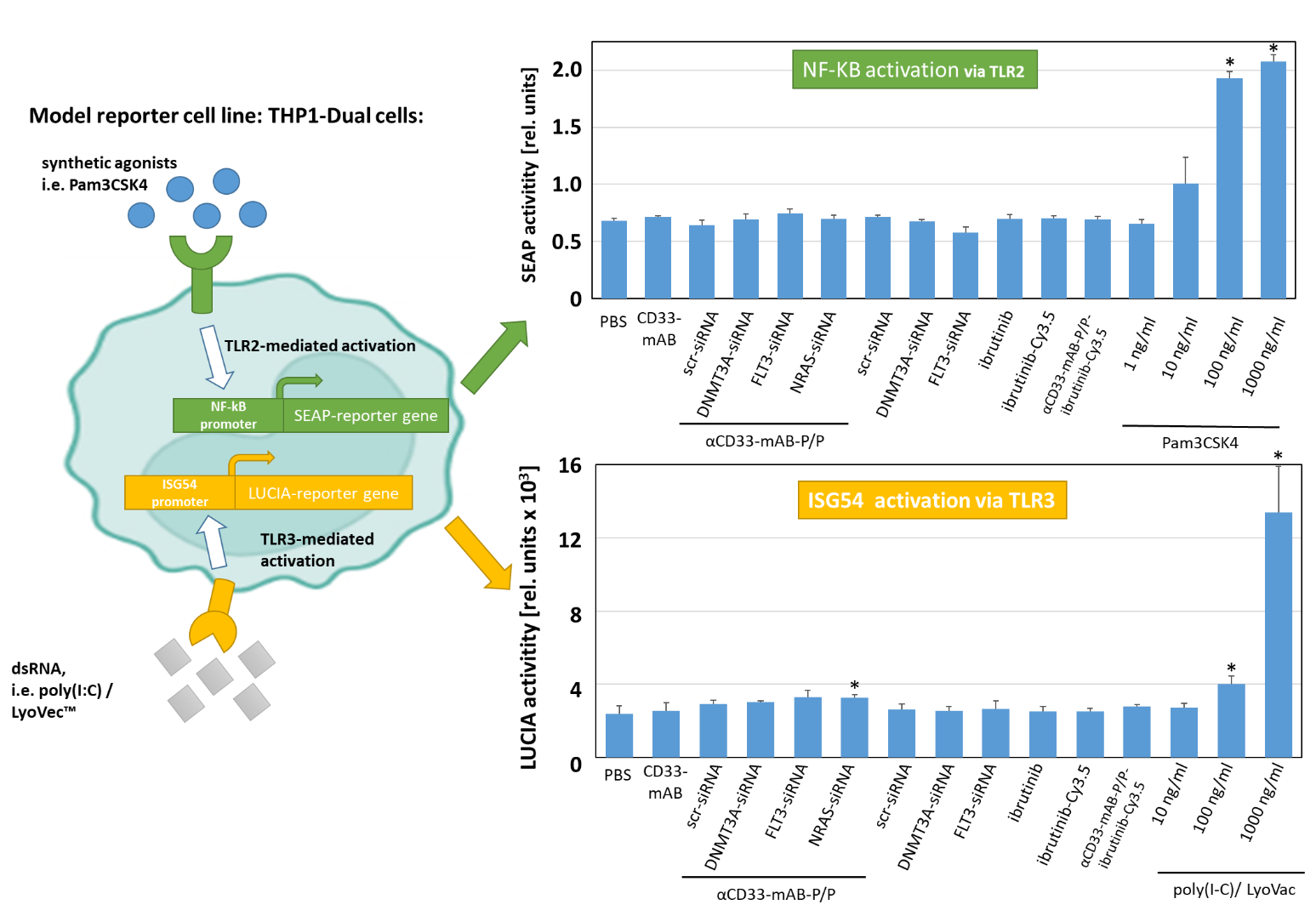


**Figure S9:** **Monitoring of NF-κB or IRF pathway activation *in vitro*.**

Left side: Schematic overview of the assay. The monocytic reporter cell line THP1-DUAL (InvivoGen) harbors 1) a secreted embryonic alkaline phosphatase (SEAP) reporter gene driven by an IFN-β minimal promoter fused to five copies of the NF-κB consensus transcriptional response element and three copies of the c-Rel binding site and 2) the Lucia gene, a secreted luciferase reporter gene, under the control of an ISG54 minimal promoter in conjunction with five IFN-stimulated response elements, which allows examination of NF-κB pathway activation via TLR2-signaling and of IRF pathway activation via TLR3 signaling. Upper right side: Only the positive control PAM3CSK4 activates the NF-κB pathway significantly (*, p<0.001, T-test). No siRNA from our panel – free or complexed in αCD33-mAB-P/P nanocarrier – or free or complexed ibrutinib-Cy3-5 activate the NF-κB pathway. Lower right side: The positive control poly(I-C)/LyoVac significantly activates the IRF-pathway (*p<0.05, T-test). Of all tested free substances, only αCD33-mAB-P/P-NRAS-siRNA nanocarrier also activates the IRF-pathway very minor (p=0.03), which might reflect a specific intrinsic reaction of the highly CD33-positive leukemic THP1 cells, which harbor an oncogenic NRAS-mutation.

**Supplemental references:**

1. Bikas A, Jensen K, Patel A, Costello J, Reynolds SM, Mendonca-Torres MC, et al. Cytochrome C Oxidase Subunit 4 (COX4): A Potential Therapeutic Target for the Treatment of Medullary Thyroid Cancer. Cancers (Basel). 2020;12(9).

2. Roh TH, Yim H, Roh J, Lee KB, Park SH, Jeong SY, et al. The loss of succinate dehydrogenase B expression is frequently identified in hemangioblastoma of the central nervous system. Sci Rep. 2019;9(1):5873.

3. Aggarwal RK, Luchtel RA, Machha V, Tischer A, Zou Y, Pradhan K, et al. Functional succinate dehydrogenase deficiency is a common adverse feature of clear cell renal cancer. Proc Natl Acad Sci U S A. 2021;118(39).

4. Chouhan S, Sawant M, Weimholt C, Luo J, Sprung RW, Terrado M, et al. TNK2/ACK1-mediated phosphorylation of ATP5F1A (ATP synthase F1 subunit alpha) selectively augments survival of prostate cancer while engendering mitochondrial vulnerability. Autophagy. 2022:1-26.

5. Wang DW, Su F, Zhang T, Yang TC, Wang HQ, Yang LJ, et al. The miR-370/UQCRC2 axis facilitates tumorigenesis by regulating epithelial-mesenchymal transition in Gastric Cancer. J Cancer. 2020;11(17):5042-55.

6. Han Y, Wu P, Wang Z, Zhang Z, Sun S, Liu J, et al. Ubiquinol-cytochrome C reductase core protein II promotes tumorigenesis by facilitating p53 degradation. EBioMedicine. 2019;40:92-105.

7. Sun Y, Xu Z, Jiang J, Xu T, Xu J, Liu P. High Expression of Succinate Dehydrogenase Subunit A Which Is Regulated by Histone Acetylation, Acts as a Good Prognostic Factor of Multiple Myeloma Patients. Front Oncol. 2020;10:563666.

8. Li ST, Huang, Shen S, Cai Y, Xing S, Wu G, et al. Myc-mediated SDHA acetylation triggers epigenetic regulation of gene expression and tumorigenesis. Nat Metab. 2020;2(3):256-69.

9. Kaneko SJ, Gerasimova T, Smith ST, Lloyd KO, Suzumori K, Young SR. CA125 and UQCRFS1 FISH studies of ovarian carcinoma. Gynecol Oncol. 2003;90(1):29-36.

10. Jun KH, Kim SY, Yoon JH, Song JH, Park WS. Amplification of the UQCRFS1 Gene in Gastric Cancers. J Gastric Cancer. 2012;12(2):73-80.

11. Wang Q, Li M, Gan Y, Jiang S, Qiao J, Zhang W, et al. Mitochondrial Protein UQCRC1 is Oncogenic and a Potential Therapeutic Target for Pancreatic Cancer. Theranostics. 2020;10(5):2141-57.

12. Han Y, Sun S, Zhao M, Zhang Z, Gong S, Gao P, et al. CYC1 Predicts Poor Prognosis in Patients with Breast Cancer. Dis Markers. 2016;2016:3528064.

13. Sato A, Takagi K, Miki Y, Yoshimura A, Hara M, Ishida T, et al. Cytochrome c1 as a favorable prognostic marker in estrogen receptor-positive breast carcinoma. Histol Histopathol. 2019;34(12):1365-75.

14. Ciccarone F, Di Leo L, Lazzarino G, Maulucci G, Di Giacinto F, Tavazzi B, et al. Aconitase 2 inhibits the proliferation of MCF-7 cells promoting mitochondrial oxidative metabolism and ROS/FoxO1-mediated autophagic response. Br J Cancer. 2020;122(2):182-93.

15. You X, Tian J, Zhang H, Guo Y, Yang J, Zhu C, et al. Loss of mitochondrial aconitase promotes colorectal cancer progression via SCD1-mediated lipid remodeling. Mol Metab. 2021;48:101203.

16. Song KH, Kim JH, Lee YH, Bae HC, Lee HJ, Woo SR, et al. Mitochondrial reprogramming via ATP5H loss promotes multimodal cancer therapy resistance. J Clin Invest. 2018;128(9):4098-114.

17. Showalter AE, Martini AC, Nierenberg D, Hosang K, Fahmi NA, Gopalan P, et al. Investigating Chaperonin-Containing TCP-1 subunit 2 as an essential component of the chaperonin complex for tumorigenesis. Sci Rep. 2020;10(1):798.

18. Ghozlan H, Showalter A, Lee E, Zhu X, Khaled AR. Chaperonin-Containing TCP1 Complex (CCT) Promotes Breast Cancer Growth Through Correlations With Key Cell Cycle Regulators. Front Oncol. 2021;11:663877.

19. Wang CH, Wang LK, Wu CC, Chen ML, Lee MC, Lin YY, et al. The Ribosomal Protein RPLP0 Mediates PLAAT4-induced Cell Cycle Arrest and Cell Apoptosis. Cell Biochem Biophys. 2019;77(3):253-60.

20. Wang YL, Zhao WW, Bai SM, Ma Y, Yin XK, Feng LL, et al. DNA damage-induced paraspeckle formation enhances DNA repair and tumor radioresistance by recruiting ribosomal protein P0. Cell Death Dis. 2022;13(8):709.
